# Water‐Soluble POSS Promotes Concurrent Coalescence and Crosslinking for Multiscale‐Reinforced, Coalescent‐Free Waterborne Coatings

**DOI:** 10.1002/advs.77495

**Published:** 2026-08-29

**Authors:** Chao Wang, Bin Wu, Lin Li, Yibo Ru, Xiaohe Zhang, Minlong Wang, Ruoyan Yang, Jie An

**Affiliations:** ^1^ Department of Nutrition and Health China Agricultural University Beijing China

**Keywords:** coalescent‐free coatings, multiscale reinforcement, interparticle crosslinking, latex coalescence, polyhedral oligomeric silsesquioxanes, waterborne coatings

## Abstract

Waterborne thermoset coatings face a fundamental formulation dilemma, whereby premature crosslinking restricts latex particle deformation and polymer diffusion, thus necessitating the use of VOC‐emitting coalescing agents to achieve continuous film formation. Here, this dilemma may be resolved using a water‐soluble, amine‐functionalized polyhedral oligomeric silsesquioxane (NPOSS) to promote cooperative coalescence and interparticle crosslinking in a latex of epoxy‐functionalized microspheres with two‐stage seeded core–shell architecture. Dissolved in water, NPOSS appeared to transiently plasticize the latex particles to facilitate coalescence while exhibiting a moderate reaction rate that permits interparticle diffusion prior to extensive crosslinking. Cryo‐TEM and DLS reveal wet‐state particle association, deformation, and fusion, while ATR‐FTIR confirms continued post‐drying network densification. This process yields a multiscale reinforcement architecture comprising a molecular‐scale covalent network, nanoscale chemical heterogeneity, and micrometer‐scale compositional homogeneity. Driven by this multiscale reinforcement, the NPOSS network simultaneously delivers enhancements in both macroscopic strength and toughness. Transparent coatings prepared without added coalescing agents or co‐solvents exhibit 2H hardness, 6.6 N scratch resistance, >89% visible transmittance, and strong chemical resistance under laboratory spotting conditions.

## Graphical abstract

Water‐soluble amine‐functionalized polyhedral oligomeric silsesquioxane promotes concurrent latex coalescence and crosslinking, allowing epoxy‐functional particles to form continuous films without added volatile coalescing agents. The resulting multiscale‐reinforced waterborne coatings combine high strength, toughness, optical transparency, scratch resistance, and chemical durability, offering a promising route toward high‐performance VOC‐free coating.

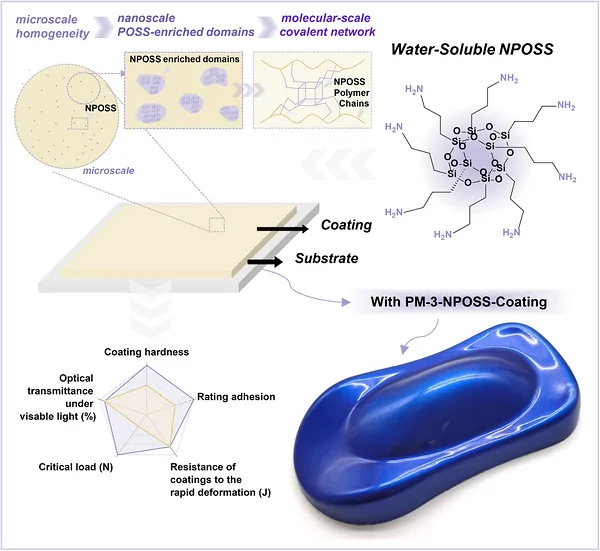

## 1 Introduction

With global demand for paints and coatings exceeding 54 million tons, the environmental burden of coating formulation remains formidable [1]. The sector consumes approximately 9.2 million tons of solvents annually worldwide, corresponding to roughly 46% of the global solvent market, making the reduction of volatile organic compounds (VOCs) a central environmental imperative of this industry [1, 2]. While waterborne coatings offer a crucial pathway to mitigating this footprint, they fail to entirely eradicate VOC emissions [3, 4]. In practice, both one‐component thermoplastic dispersions and many 2K thermosetting systems (two‐component system in which the resin and curing agent are stored separately and mixed prior to application) still frequently rely on volatile coalescing agents or co‐solvents to achieve continuous film formation. Indeed, standard waterborne coatings can contain VOC levels up to 250 g/L [5, 6]. Furthermore, despite rapid commercial adoption, it remains widely recognized that waterborne coatings continue to underperform relative to their solvent‐borne counterparts in the most demanding, high‐performance applications [5, 6].

The challenge arises because waterborne and solventborne coatings follow fundamentally different film‐formation pathways [6, 7, 8]. In particulate waterborne coatings, the polymer is present as latex particles rather than as molecularly dissolved chains, and continuous‐film formation therefore proceeds through a drying‐induced coalescence sequence, involving water evaporation, particle concentration and packing, capillary‐pressure‐driven particle deformation, and ultimately polymer interdiffusion across interparticle boundaries until discrete particle identities disappear [6, 7, 8, 9]. To facilitate this process under practical drying conditions, volatile coalescing agents, such as dipropylene glycol methyl ether and dipropylene glycol *n*‐butyl ether, have to be added to temporarily depress the effective glass transition temperature and minimum film formation temperature [7, 8, 10]. More recently, alternative strategies, including reactive coalescing agents, nonvolatile plasticizers and formulation‐optimized waterborne 2K coatings, have been explored to reduce this dependence [11, 12, 13, 14]. Nevertheless, these approaches often remain limited by the use of volatile auxiliary organic components, the retention of permanently softened domains or the use of the specific formulation, leaving coalescent‐free film formation in high‐performance systems unresolved [6, 12, 13, 14].

This challenge becomes even more acute in waterborne thermosets, where drying‐induced physical coalescence is inseparably coupled with covalent network formation [15, 16, 17, 18]. As Winnik and co‐workers have emphasized, film strength develops only when polymer chains bridge the interfaces between adjacent particles, implying that substantial interparticle diffusion must occur before extensive crosslinking immobilizes the system [17, 18, 19, 20, 21]. If curing proceeds too early, network formation remains largely confined within individual particles, interfacial healing is arrested, and the resulting films suffer from weak interparticle adhesion [16]. Conversely, if curing is delayed sufficiently to preserve interfacial mobility, continuous film formation must still rely primarily on drying‐driven particle deformation and chain interdiffusion, which in practice often maintains the dependence on VOC‐emitting coalescing agents [10, 11, 14, 15, 21].

Most existing 2K waterborne thermoset strategies resolve this contradiction by delaying curing. This is often evident in 2K waterborne polyurethane systems, which remain the dominant industrial platform for high‐performance waterborne thermosets [22, 23, 24, 25]. In these formulations, hydrophilically modified polyisocyanates (hmPICs) are typically introduced as a separate dispersed reactive phase rather than as molecularly dissolved crosslinkers. Typically, the polyisocyanate component is diluted with about 30 wt % organic solvent to reduce viscosity, and forms droplets of about 20 nm when dispersed in water [26]. Because the curing phase is emulsified rather than dissolved, the probability of reaction between dispersed hmPIC droplets and polymer particles remains limited before extensive water removal, and polymer diffusion in the film can proceed on a faster timescale than network formation [22, 23, 26]. A similar logic underlies diacetone acrylamide / adipic dihydrazide‐based waterborne crosslinking systems [27, 28, 29, 30, 31]. The reaction between carbonyl groups and hydrazides is favored only after water evaporation and during film formation [27], because the condensation between ketone and hydrazide groups is governed by a water‐dependent equilibrium [29]. Consequently, irreversible network formation occurs mainly upon dehydration [30]. While both of these widely used strategies effectively control the coalescence‐curing sequence to yield dense thermoset films, their fundamental reliance on post‐drying physical deformation dictates that they cannot eliminate the need for co‐solvents or volatile coalescing agents [30, 32]. Herein, we hypothesize that identifying a water‐soluble curing agent capable of transiently plasticizing latex particles to facilitate coalescence, while exhibiting moderated reactivity to allow interparticle diffusion before extensive crosslinking, could provide a route to fundamentally eliminating the reliance of waterborne coatings on VOC‐emitting coalescing agents [10, 11]. In this context, polyhedral oligomeric silsesquioxane (POSS) is particularly attractive. POSS is a molecular organic–inorganic hybrid featuring a rigid silsesquioxane cage that can be functionalized with various organic functional groups [33, 34, 35, 36, 37, 38, 39, 40]. In selected polymer systems, dissolved POSS has been associated with increased free volume and enhanced chain mobility, suggesting that it could promote film formation in a manner similar to that of a coalescing agent [35, 37, 41, 42, 43]. Furthermore, modifying the organic groups on POSS not only provides the reactive sites necessary for curing but also tunes its aqueous solubility [44].

**SCHEME 1:**
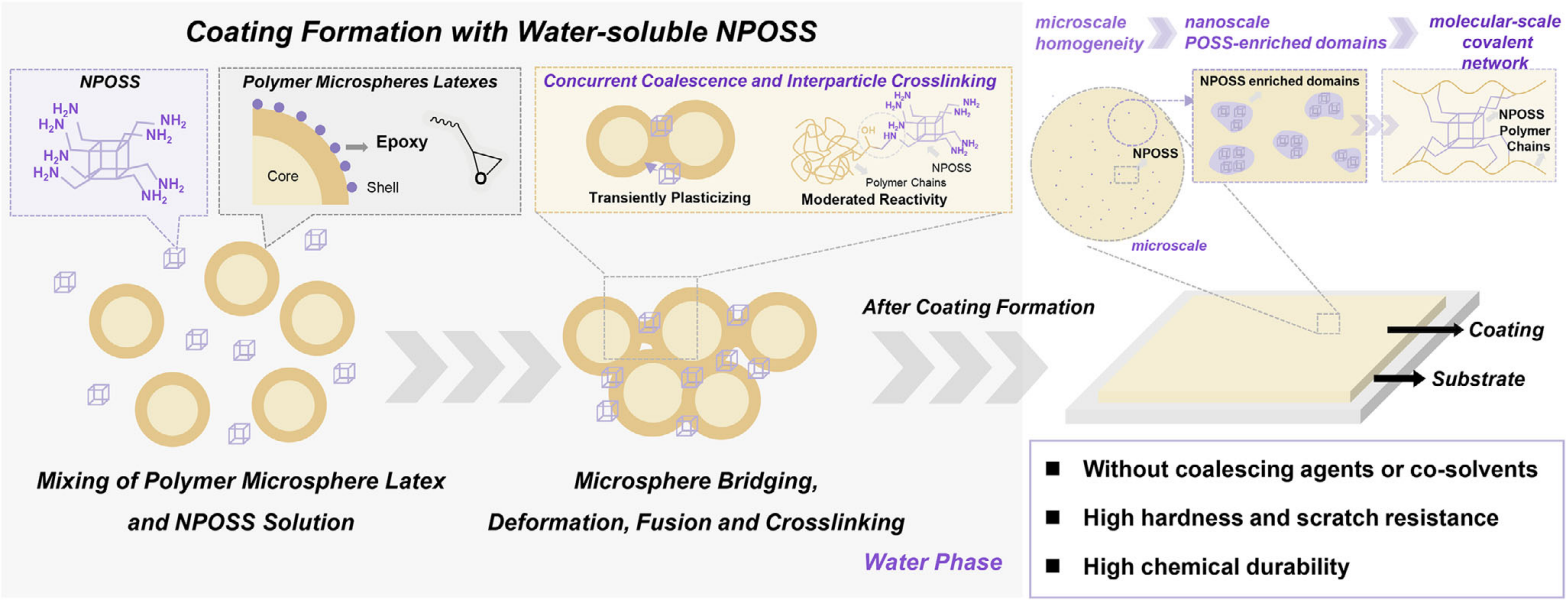
Proposed Water‐soluble NPOSS‐promoted concurrent coalescence and interparticle crosslinking for coalescent‐free high‐performance waterborne thermoset coatings.

To realize this concept, we desired a water‐soluble, amine‐functionalized POSS (NPOSS). We tested our hypothesis using a latex of epoxy‐functionalized polymer microspheres featuring two‐stage seeded core–shell architecture, with NPOSS serving as the curing agent. As shown in Scheme 1, we demonstrate that in this 2K system, NPOSS enables concurrent coalescence and interparticle crosslinking. Cryo‐TEM directly visualizes microsphere bridging, deformation, and fusion before bulk water removal is complete, whereas ATR‐FTIR confirms continued post‐drying curing. The resulting films develop a multiscale reinforcement architecture comprising a molecular‐scale covalent network, nanoscale POSS‐associated heterogeneous nanostructures, and micrometer‐scale compositional homogeneity, as characterized by SAXS and SEM‐EDS. Driven by this multiscale reinforcement, the NPOSS network results in mechanical properties that are consistent with circumventing the strength–toughness trade‐off that typically limits conventional curing agents, while transparent coatings prepared without added coalescing agents or co‐solvents exhibit high hardness, scratch resistance, optical clarity, and chemical durability. These results suggest 2K coating using water‐soluble POSS as a promising route to coalescent‐free high‐performance waterborne thermoset coatings.

## 2 Results and Discussion

### 2.1 Preparation of Epoxy‐Functionalized Microsphere Latexes With Two‐Stage Seeded Core–Shell Architecture

**FIGURE 1:**
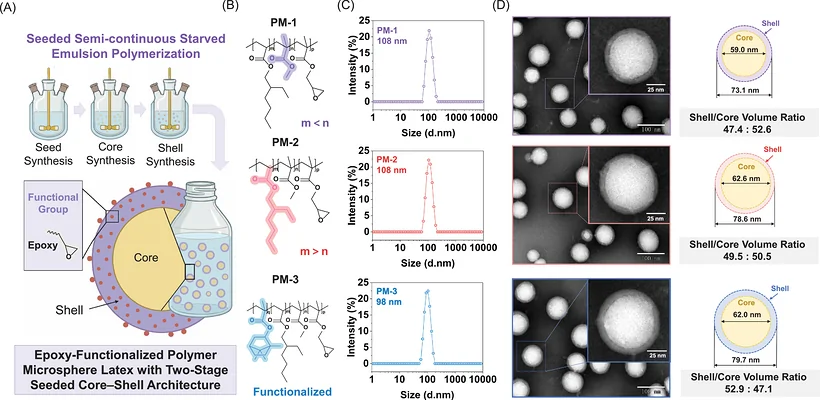
Design, synthesis, and characterization of epoxy‐functionalized polymer microsphere latex with two‐stage seeded core–shell architecture. (A) The preparation of epoxy‐functionalized polymer microsphere latexes with two‐stage seeded core–shell architecture via seeded semi‐continuous starved emulsion polymerization. (B) Nominal two‐stage particle designs of the three polymer microspheres (PM‐1, PM‐2, and PM‐3), as defined by the programmed monomer feeds. (C) DLS intensity‐weighted particle size distributions of PM‐1, PM‐2, and PM‐3. (D) Representative TEM images showing the microspheres morphology at low magnification (bottom; scale bars: 100 nm) and magnified views of typical individual microspheres (top; scale bars: 25 nm). For geometric comparison only, the contrast‐defined inner and outer regions are represented as nominal concentric core and shell domains. Under this nominal geometry, the core volume was calculated as Equation (1): $V_{core}=\frac{4}{3}\pi r^{3}$ (1) and the shell volume as Equation (2): $V_{shell}=\frac{4}{3}\pi \left(R^{3}-r^{3}\right)$ (2), where r is the nominal inner‐region radius and R is the overall particle radius.

To evaluate NPOSS‐associated coalescence and crosslinking, three latexes with intended two‐stage seeded core–shell architecture were synthesized by seeded emulsion polymerization using a starved‐feed semicontinuous monomer‐addition strategy [45], in which epoxy‐functional monomers were introduced during the second‐stage feed (Figure 1A,B and Table 1). The compositions of these latexes were selected to reflect the diverse performance requirements encountered in coating applications [6, 46, 47, 48]. For instance, applications requiring high substrate conformability, such as coatings for leather and textiles, generally favor low T_g_ latexes [47, 48]. By contrast, protective applications for appliances and automotive components generally employ higher‐Tg polymers when greater coating hardness is required [49, 50]. To capture these representative industrial requirements, three distinct latex systems were formulated. PM‐1 was designed with a higher fraction of hard monomers, whereas PM‐2 contained a higher fraction of soft monomers to enhance flexibility. PM‐3 was derived from PM‐1 by incorporating isobornyl methacrylate (IBOMA) [51], a monomer widely used in industrial formulations to balance the thermal and mechanical properties, while the surfactant was changed from CTAB to TTAB to accommodate the corresponding change in monomer composition.

**TABLE 1:** Composition and characterization of epoxy‐functionalized microsphere latexes with two‐stage seeded core–shell architecture.

Entry	First‐stage Monomer	Second‐stage Monomer	Surfactant	Size / nm	PDI	M_w_ / g/mol	M_n_ / g/mol	Đ
PM‐1	MMA 2‐EHA	MMA 2‐EHA GMA	CTAB ER‐30	108	0.048	1.3×10^5^	6.2×10^4^	2.03
PM‐2	MMA 2‐EHA	MMA 2‐EHA GMA	CTAB ER‐30	108	0.041	1.3×10^5^	6.3×10^4^	1.98
PM‐3	MMA 2‐EHA	MMA 2‐EHA GMA IBOMA	TTAB ER‐30	98	0.064	1.1×10^5^	5.9×10^4^	1.87

Under the seeded emulsion polymerization conditions, all three formulations afforded stable milky latexes with solids content of 37.7–38.0 wt.% (Table S1). Dynamic light scattering revealed unimodal particle‐size distributions with hydrodynamic diameters (Figure 1C and Table 1) of 108 nm (PM‐1), 108 nm (PM‐2), and 98 nm (PM‐3), PDIs below 0.07, and zeta potentials above 34.0 mV (Table S1), in line with colloidally stable dispersions. Negative‐stain TEM images further exhibited intraparticle bright/dark contrast for all three samples (Figure 1D). The estimated shell domains volume fraction from representative particles is approximately 50%, which corresponds closely to the two‐stage monomer feed ratio (approximately 1:1). Gel permeation chromatography (GPC) of the THF‐soluble fractions showed Mw above 1.1 × 10^5^ g/mol and Mn above 5.8 × 10^4^ g/mol (Table 1 and Figure S1). The THF‐insoluble fractions (gel contents) of the freeze‐dried latex powders, determined by Soxhlet extraction, were summarized in Table S1 and ranged from approximately 25% to 28% across the three formulations. Differential scanning calorimetry (DSC) of the freeze‐dried latex powders revealed glass transition temperatures (Tg) of approximately 41, −15, and 36°C for PM‐1, PM‐2, and PM‐3, respectively (Figure S2). The single broad Tg observed for freeze‐dried PM‐1 latex solids (Figure S2) reflects the dried‐state thermal response after freezing, dehydration, and particle packing, and therefore was not used as direct evidence to confirm or exclude a discrete compositional core–shell structure.

To further evaluate the long‐term stability of the epoxy latex in water, we stored the PM‐3 latex at room temperature for one year and then re‐examined its properties. As a representative example, the PM‐3 epoxy latex exhibited no appreciable changes in particle size, zeta potential (Table S2), or the characteristic epoxideband near 910 cm^−1^ in the FTIR spectra (Figure S3), indicating favorable storage stability (see Section S3 in the Supporting Information for details).

### 2.2 Time‐Resolved Characterization of NPOSS‐Promoted Coalescence and Interparticle Crosslinking in Epoxy‐Functionalized Microsphere Latex

**FIGURE 2:**
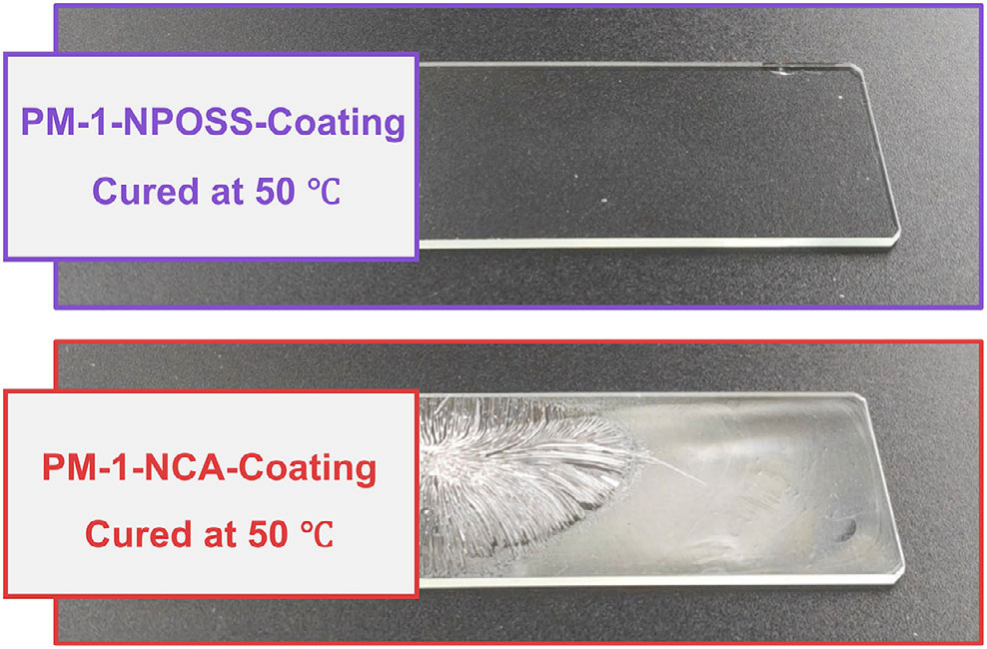
Comparison of the film‐forming performance of PM‐1 at 50°C with and without NPOSS.

To elucidate the process of NPOSS‐enabled coalescence and interparticle crosslinking, PM‐1, which possesses a relatively high glass transition temperature (Tg = 41°C, Figure S2), was selected as the model latex. Compared with PM‐2 and PM‐3, its higher T_g_ presents a more stringent case for film formation. At 50°C, in the absence of external coalescing agents, neat PM‐1 failed to form a continuous film, yielding only a whitish, brittle, and fragmented coating upon water evaporation. However, upon the addition of an aqueous NPOSS solution (2.0 mL, 31.60 mg/mL, at an amine‐to‐epoxy equivalent ratio of 2:1) to the PM‐1 latex (1.0 g), the cast layers successfully yielded optically continuous and transparent films at the same temperature (Figure 2). Further experiments were therefore performed to elucidate the corresponding film‐formation pathway.

**FIGURE 3:**
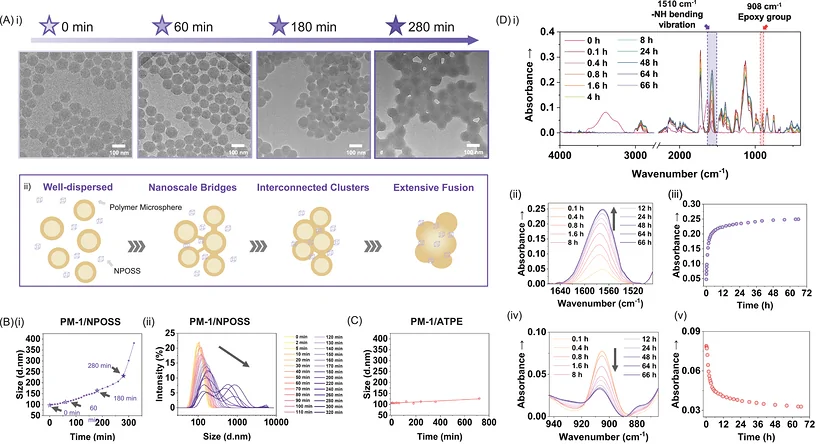
NPOSS‐enabled coalescence and interparticle crosslinking in epoxy‐functionalized microsphere latex with two‐stage seeded core–shell architecture. (A) Morphological evolution of the PM‐1 latex after the addition of NPOSS. (i) Cryo‐TEM images collected at 0, 60, 180, and 280 min. Scale bars: 100 nm. (ii) Schematic illustration of the corresponding structural evolution from well‐dispersed particles to nanoscale bridges, interconnected clusters, and extensive fusion. (B) DLS monitoring of the PM‐1/NPOSS system. (i) Time‐dependent change in the average particle size after NPOSS addition. (ii) Evolution of the intensity‐weighted particle size distribution with time. (C) Time‐dependent change in particle size for the PM‐1/ATPE system after ATPE addition, shown as a control. (D) In situ ATR‐FTIR monitoring of the PM‐1/NPOSS system. (i) Time‐resolved FTIR spectra, in which the band at 1510 cm^−1^ is assigned to the N–H bending vibration and the band at 908 cm^−1^ to the epoxy group. (ii) Enlarged view of the 1510 cm^−1^ region. (iii) Time‐dependent absorbance at 1510 cm^−1^. (iv) Enlarged view of the 908 cm^−1^ region. (v) Time‐dependent absorbance at 908 cm^−1^.

Cryogenic transmission electron microscopy (cryo‐TEM) was initially employed to monitor the microstructural evolution of the wet formulation following the addition of NPOSS. After mixing, the dispersion was kept at room temperature, and aliquots were withdrawn at defined time intervals for cryo‐TEM observation (Figure 3A); thus, the structural evolution prior to freezing was followed under room‐temperature conditions. Immediately after mixing, the PM‐1 microspheres remained discrete, nearly spherical, and well‐dispersed. After 60 min, nanoscale filamentous bridges were observed between adjacent microspheres. These features potentially arose from localized adhesive contacts followed by Brownian‐motion‐induced relative displacement of partially attached particles, resulting in a drawn filament‐like morphology [52, 53, 54]. However, the static nature of cryo‐TEM precludes direct confirmation of the proposed dynamic pathway. By 180 min, these fibrillar connections had evolved into interconnected clusters, and slight particle deformation became evident, although the contours of individual particles were still recognizable. After 280 min, the original particle boundaries had nearly disappeared, indicating extensive fusion of the microspheres.

Because cryo‐TEM probes only localized regions, DLS was further used to track the temporal evolution of the particle size within the bulk mixture (2.0 mL of 31.60 mg/mL aqueous NPOSS solution added to 1.0 g of PM‐1). As in the cryo‐TEM experiments, the sample was mixed at room temperature and then monitored at room temperature throughout the measurement. The DLS results were highly consistent with the cryo‐TEM observations (Figure 3B). The initial hydrodynamic diameter of the neat latex was approximately 100 nm. Over the subsequent 240 min post‐mixing, the apparent diameter of the wet formulation increased at a relatively constant rate to about 200 nm, maintaining a unimodal, near‐normal size distribution. After 240 min, however, the mean particle size increased more rapidly, and the distribution became bimodal, in good agreement with the pronounced fusion observed by cryo‐TEM at 280 min. These results indicate that NPOSS can induce particle association, deformation, and interparticle diffusion before complete water removal. It should be noted that DLS requires sample dilution, which does not replicate the concentrated conditions of film formation. The DLS data are therefore interpreted primarily for qualitative comparison between formulations.

To determine whether the observed microsphere fusion originated from the POSS framework itself or simply from amine–epoxy reactions between NPOSS and the latex particles, another water‐soluble multifunctional curing agent, polyetheramine (ATPE), was examined as a control. ATPE contains polypropylene glycol segments that can act as coalescing agents, and their terminal ‐NH_2_ groups can crosslink with epoxides similarly to NPOSS [55]. Nevertheless, DLS measurements showed that, after mixing polyetheramine with PM‐1 under comparable conditions (2.0 mL of a 37.15 mg/mL aqueous polyetheramine solution added to 1.0 g of PM‐1, at the same amine equivalent as NPOSS), no significant increase in particle size occurred over 720 min (Figure 3C). This comparison indicates that the microsphere fusion observed in the NPOSS‐containing system cannot be ascribed solely to amine–epoxy crosslinking, but is more plausibly associated with the POSS moiety itself. A reasonable interpretation is that dissolved NPOSS may partition from the aqueous phase into the latex particles and may facilitate chain mobility, thereby promoting interparticle adhesion and fusion [36, 41].

To further probe the amine–epoxy reaction after mixing NPOSS with the latex, In situ ATR‐FTIR measurements were performed under conditions that more closely resemble practical coating application. A drop of the mixture of NPOSS (2.0 mL of a 31.6 mg/mL aqueous solution) and PM‐1 latex (1.0 g) was cast directly onto the ATR crystal, and the In situ measurement was carried out at 50°C. After approximately 10 min, once most of the water had evaporated, the evolution of the amine‐ (1510 cm^−1^) and epoxide‐related (908 cm^−1^) signals could be monitored In situ. The spectroscopic data revealed a moderate reaction rate (Figure 3D). The epoxy–amine reaction proceeded relatively rapidly during the first 6 h, followed by a much slower stage in which residual functional groups were gradually consumed. The reaction approached completion after approximately 66 h.

These data indicate that curing is initiated soon after mixing and remains active for more than 6 h under the post‐casting condition monitored at 50°C. In parallel, cryo‐TEM and DLS show that particle association and fusion occur within several hours under room‐temperature wet‐state conditions. Under practical coating conditions, ongoing water loss would further promote particle packing, while the resulting capillary forces should accelerate particle deformation and fusion. It is therefore reasonable to infer that, during actual film formation, particle coalescence occurs on a timescale substantially shorter than 5 h, whereas curing continues for some time after substantial polymer diffusion has already taken place. According to the framework proposed by Winnik and co‐workers, such a temporal sequence is highly favorable for the formation of mechanically robust, high‐performance coatings [17, 19, 22].

### 2.3 Characterization of the Multiscale Structural Architecture of NPOSS/Latex Films

**FIGURE 4:**
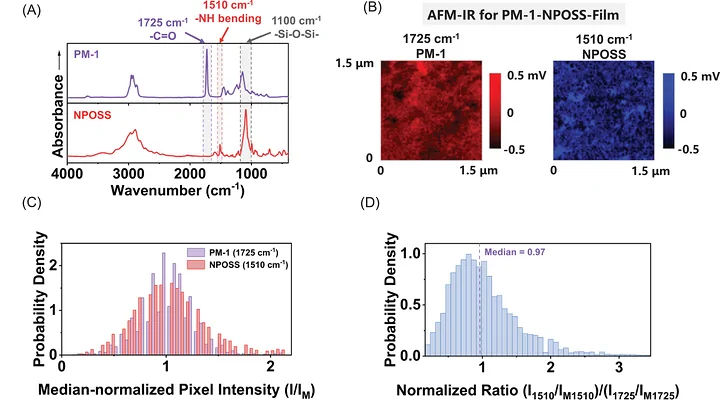
AFM‐IR analysis of the PM‐1‐NPOSS‐Film. (A) Reference ATR‐FTIR spectra of PM‐1 and NPOSS used for AFM‐IR band assignment. The band at 1725 cm^−1^ was assigned to the ester C═O stretching vibration of the acrylic matrix, while the band at 1510 cm^−1^ was assigned to an amine‐associated vibration. (B) AFM‐IR intensity maps recorded at 1725 and 1510 cm^−1^ from the same cross‐sectional region of PM‐1‐NPOSS‐Film. (C) Median‐normalized pixel‐intensity distributions extracted from the 1725 and 1510 cm^−1^ AFM‐IR maps. The coefficients of variation were 0.26 for the 1725 cm^−1^ matrix‐reference signal and 0.35 for the 1510 cm^−1^ amine‐associated signal. (D) Probability‐density distribution of the pixel‐wise normalized intensity ratio, calculated as (I_1510_/I_M1510_)/(I_1725_/I_M1725_), where I_M_ is the median intensity of the corresponding map. The dashed line indicates the median ratio value of 0.97.

The NPOSS‐cured film (PM‐1‐NPOSS‐Film) derived from PM‐1 was selected as the model material for structural characterization. AFM‐IR, SAXS, and SEM‐EDS collectively revealed a structural architecture characterized by nanoscale chemical heterogeneity and micrometer‐scale compositional homogeneity. AFM‐IR mapping was performed on film cross‐sections to resolve the nanoscale chemical distribution. The absorption at 1725 cm^−1^, assigned to the ester C═O stretching vibration of the (meth)acrylate segments in the latex polymer, was mapped together with the band at 1510 cm^−1^, which is attributed to amine‐related vibrations associated with NPOSS (Figure 4A). The maps showed that the original core–shell morphology had been largely erased throughout the film interior, and no discernible interparticle boundaries remained, indicating extensive interparticle diffusion and effective particle fusion during film formation (Figure 4B). To support the AFM‐IR interpretation more quantitatively, pixel‐intensity distributions were extracted from the 1725 and 1510 cm^−1^ maps acquired over the same region (see Section S4 in the SI for the details). After median normalization, both maps showed broadened intensity distributions, indicating spatially non‐uniform chemical responses (Figure 4C). The 1510 cm^−1^ signal exhibited a broader distribution than the 1725 cm^−1^ reference signal, with coefficients of variation values of 0.35 and 0.26, respectively. The normalized I_1510_/I_1725_ ratio distribution was centered near unity with a median of 0.97 but displayed a right‐hand tail, suggesting localized enhancement of the amine‐associated response relative to the acrylic matrix (Figure 4D). Nevertheless, the AFM‐IR maps revealed the presence of distinct chemical heterogeneity, manifested as NPOSS‐associated heterogeneous nanostructures interspersed within the polymer matrix [56, 57].

**FIGURE 5:**
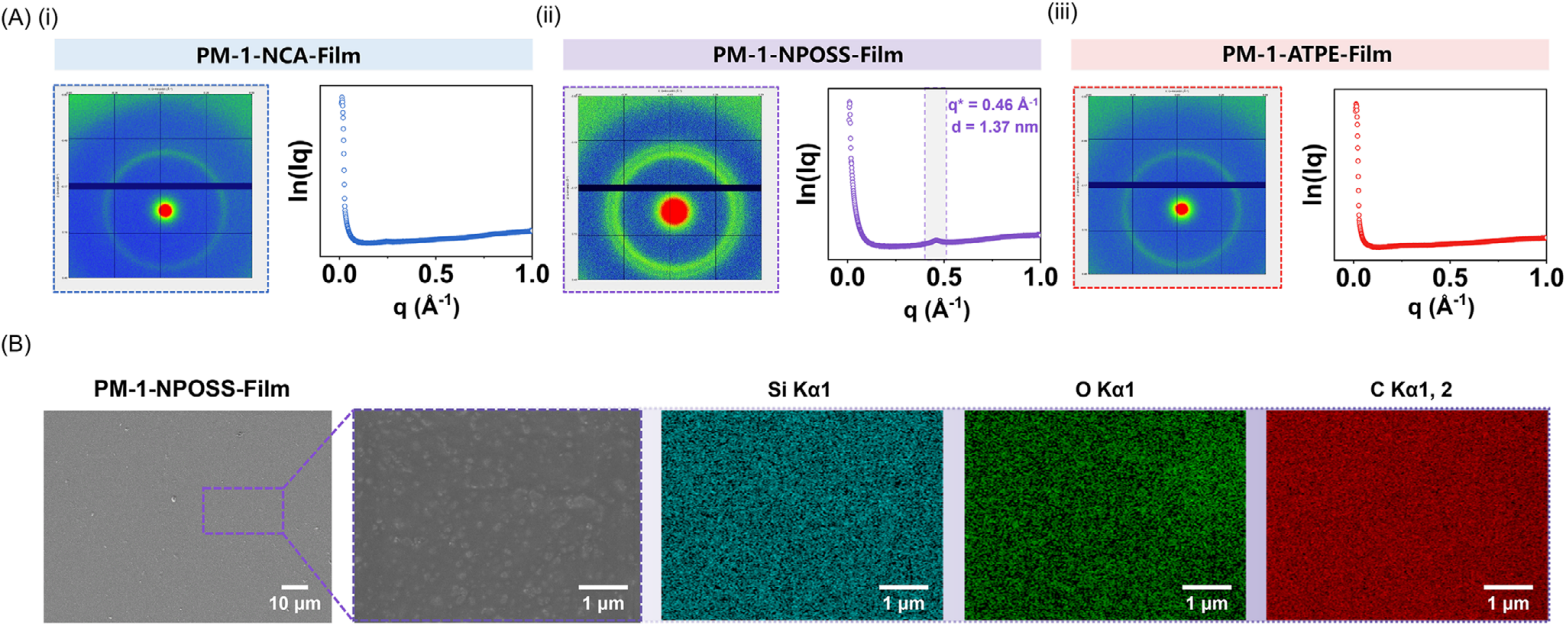
SAXS and SEM‐EDS Characterization of the PM‐1‐NPOSS‐Film. (A) SAXS characterization of PM‐1‐NCA‐Film, PM‐1‐NPOSS‐Film, and PM‐1‐ATPE‐Film. (i) Two‐dimensional SAXS patterns of the three films. (ii) Corresponding one‐dimensional SAXS profiles plotted as ln[I(q)] versus q. A weak broad scattering feature is observed for PM‐1‐NPOSS‐Film at q* = 0.46 Å^−1^, corresponding to a characteristic spacing of d = 1.37 nm. (B) SEM images and EDS elemental mapping of PM‐1‐NPOSS‐Film, showing (i) a low‐magnification SEM image, (ii) an enlarged view of the boxed region, and the distributions of (iii) Si, (iv) O, and (v) C. Scale bars: 10 µm in (i) and 1 µm in (ii)–(v).

To further probe this structural heterogeneity, SAXS measurements were performed on the cured films (Figure 5A and Figure S4 in the SI). The NPOSS‐cured samples (PM‐1‐NPOSS‐Film, PM‐2‐NPOSS‐Film and PM‐3‐NPOSS‐Film) all exhibited a weak, broad scattering feature centered at q* = 0.45 – 0.46 Å^−1^, corresponding to a characteristic spacing of d = 1.37 – 1.40 nm. Because no sharp peaks or higher‐order reflections were observed, this feature is more appropriately interpreted as a short‐range structural correlation rather than a long‐range ordered nanostructure. Such a scattering response is suggestive of heterogeneous nanostructure. However, because chemically heterogeneous nanostructures have also been reported in amine‐cured epoxy networks as a consequence of spatially inhomogeneous curing [58, 59], the origin of the present feature was not immediately evident. To clarify this point, SAXS measurements were also conducted on a fragmented neat PM‐1 film prepared without curing agent or coalescing agent (PM‐1‐NCA‐Film, NCA = no curing agent) and on a PM‐1 film cured with ATPE (PM‐1‐ATPE‐Film). Neither control sample displayed a comparable broad scattering feature, suggesting this feature is associated with NPOSS incorporation. This comparison is consistent with the interpretation that the observed scattering feature arises from NPOSS‐associated structural correlations rather than from amine–epoxy curing alone.

The low‐q region was further analyzed using Guinier and Debye–Bueche descriptions to estimate the larger‐scale electron‐density heterogeneity (Figure S5 and Table S3). The Guinier analysis yielded apparent (R_g_) values of approximately 13.1–22.3 nm, while the Debye–Bueche analysis gave correlation lengths (ξ) of approximately 8.8–9.7 nm for the NPOSS‐containing films. These length scales are substantially larger than the molecular dimension of a single T8 POSS cage, suggesting that the SAXS contrast arises from NPOSS‐associated correlated regions rather than isolated single cages. However, because SAXS is not chemically specific, these features are described as NPOSS‐associated short‐range structural correlations that contribute to nanoscale heterogeneity.

Complementary to these nanoscopic findings, SEM‐EDS analysis revealed no evidence of micrometer‐scale heterogeneity (Figure 5B). The film cross‐sections appeared uniform. Elemental Si mapping showed a highly homogeneous silicon distribution without detectable macroscopic aggregates, while C and O distributions also remained spatially isotropic. The NPOSS‐cured film is therefore more accurately described as nanoscopically heterogeneous yet microscopically uniform.

### 2.4 Mechanical and Thermal Properties of NPOSS/Latex Films

**FIGURE 6:**
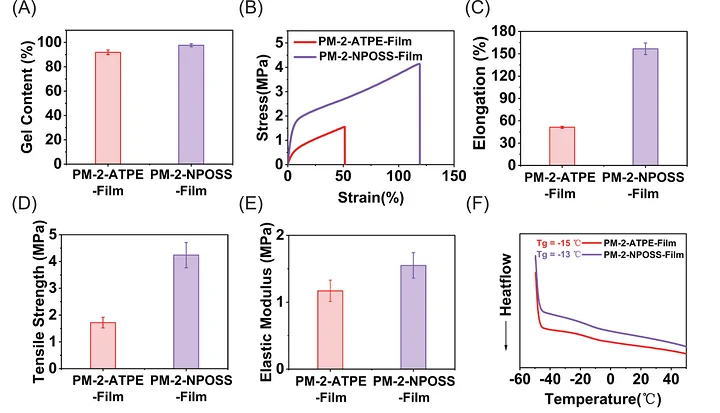
The gel content and thermo‐mechanical properties of PM‐2 films. (A) Gel contents of PM‐2‐ATPE‐Film and PM‐2‐NPOSS‐Film. (B) Representative tensile stress–strain curves of PM‐2‐ATPE‐Film and PM‐2‐NPOSS‐Film. (C) Elongation at break. (D) Tensile strength. (E) Elastic modulus. Gel content (A) and mechanical properties (C–E) were each measured in triplicate as mean ± SD (n = 3 specimens per formulation). (F) DSC thermograms of PM‐2‐NCA‐Film, PM‐2‐ATPE‐Film, and PM‐2‐NPOSS‐Film, obtained from one specimen per formulation (n = 1); the reported Tg values were individual measurements without statistical error estimates.

To evaluate the impact of NPOSS on the macroscopic performance of the coatings, the mechanical and thermal properties of the films were systematically investigated. Because the balance of stiffness and extensibility is particularly important for coatings on compliant substrates, the lower‐Tg PM‐2 latex was selected as the model material. To distinguish the specific contributions of the POSS architecture from those of amine‐based crosslinking, two control groups were established: a neat PM‐2 film without curing agents (PM‐2‐NCA‐Film) and a PM‐2 film crosslinked with an equivalent amount of ATPE (PM‐2‐ATPE‐Film). To minimize the influence of differences in overall network formation, the gel contents of the cured samples were first measured. The results indicated that both systems achieved high levels of curing (95.6 ± 1.2% for PM‐2‐NPOSS‐Film and 90.6 ± 2.9% for PM‐2‐ATPE‐Film, mean ± standard deviation, n = 3 specimens per formulation; Figure 6A), with the gel content of PM‐2‐NPOSS‐Film being moderately higher but still comparable to that of PM‐2‐ATPE‐Film. The differences observed in the subsequent mechanical and thermal measurements are therefore unlikely to arise solely from a large difference in crosslink density. Meanwhile, chloride titration indicated that approximately 77.4% of the amine groups in NPOSS are protonated (Table S4). This protonation degree did not significantly impede the curing reaction, as reflected by a final gel content of 95.6% for the PM‐2‐NPOSS‐Film.

Mechanical testing showed that PM‐2‐NPOSS‐Film exhibited simultaneous increases in elongation at break, tensile strength, and elastic modulus relative to PM‐2‐ATPE‐Film (Figure 6B). The elongation at break increased from 51.3 ± 1.4% to 157 ± 8% (Figure 6C), the tensile strength increased from 1.7 ± 0.2 MPa to 4.2 ± 0.5 MPa (Figure 6D), and the elastic modulus increased from 1.2 ± 0.2 MPa to 1.6 ± 0.2 MPa (Figure 6E). Such concurrent improvements in stiffness, strength, and extensibility are notable for thermosetting films and suggest that the improved performance of PM‐2‐NPOSS‐Film cannot be explained by crosslink density alone. Instead, the improvement is more plausibly attributed to the combined effects of reinforcement by the rigid silsesquioxane cage and the multiscale structural architecture identified above. First, the rigid inorganic POSS cages may serve as high‐functionality hybrid crosslinking centers that effectively distribute stress within the polymer matrix. Second, the nanoscale chemical heterogeneity observed in Section 2.3 may play a pivotal role in energy dissipation. These NPOSS‐associated heterogeneous nanostructures may act as reinforcing clusters, and may provide rigid heterogeneous junctions or tortuous paths that arrest crack propagation and trigger localized shear‐yielding. This potentially prevents the catastrophic brittle failure typically observed in densely crosslinked homogeneous networks, thereby enabling higher extensibility alongside increased strength [43, 60, 61].

DSC yielded Tg values of approximately −15, −15, and −13°C for PM‐2, PM‐2‐ATPE‐Film, and PM‐2‐NPOSS‐Film (Figure 6F and Figure S2). Notably, incorporation of the cross‐linkers did not lead to a pronounced increase in Tg. A similarly modest shift was observed for PM‐1 (Tg = 41°C) versus PM‐1‐NPOSS‐Film (Tg = 41°C) and for PM‐3 (Tg = 36°C) versus PM‐3‐NPOSS‐Film (Tg = 39°C).

### 2.5 Laboratory‐Scale Evaluation of NPOSS/Latex for High‐Performance Clearcoat Applications

To assess selected coating properties at the laboratory scale, both PM‐3‐NPOSS‐Coating and PM‐3‐ATPE‐Coating were spray‐coated onto standard aluminum panels and a pre‐primed automotive model substrate, yielding dry‐film thicknesses of 11.5 ± 0.6 µm and 11.7 ± 2.7 µm (Table S5). Automotive clearcoats serve as the outermost protective barrier and must deliver exceptional gloss and transparency while simultaneously resisting mechanical scratches and environmental weathering. Consequently, they represent a particularly demanding target for waterborne coatings [62]. Achieving a balance between aesthetic excellence and mechanical robustness remains a formidable challenge [5]; notably, current U.S. EPA benchmarks for automobile assembly still rely on waterborne basecoats paired with solvent‐borne clearcoats [63].

**FIGURE 7:**
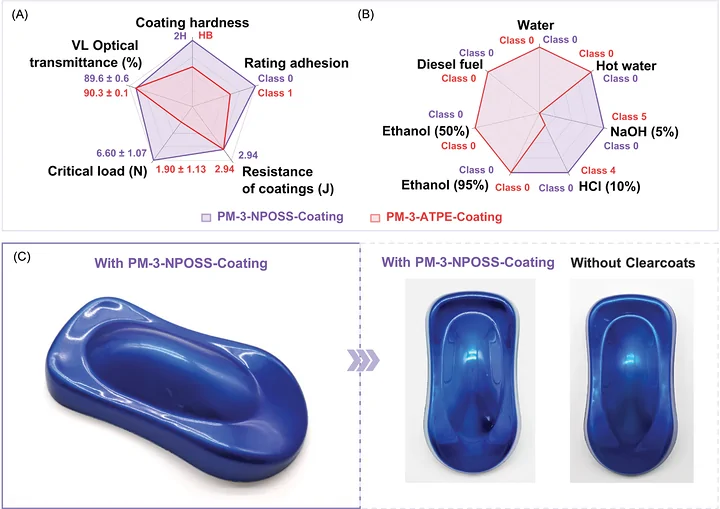
Properties and applications of PM‐3‐based coatings. Quantitative continuous data are presented as mean ± SD from three separately coated panels (n = 3). Pencil hardness, cross‐cut adhesion, impact resistance, and chemical‐resistance classifications were each evaluated on three coated panels per formulation; the plotted categorical values represent the results consistently obtained across the three panels or the lowest rating. (A) Comparison of the overall performance of PM‐3‐NPOSS‐Coating and PM‐3‐ATPE‐Coating, including coating hardness, adhesion rating, resistance to rapid deformation, critical load, and optical transmittance in the visible region. (B) Comparison of the chemical resistance of PM‐3‐NPOSS‐Coating and PM‐3‐ATPE‐Coating against water, hot water, 5% NaOH solution, 10% hydrochloric acid solution, 95% ethanol, 50% ethanol, and diesel fuel. (C) Laboratory‐scale demonstration of PM‐3‐NPOSS‐Coating as a clearcoat on an automotive model substrate. The left panel shows the coated sample in an oblique view, and the right panel compares the top views of samples with and without the coating.

Motivated by this challenge, the performance of our coalescent‐free PM‐3‐NPOSS‐Coating was evaluated using ISO‐standard protocols for clear topcoats. All coating‐performance measurements described below were conducted using three separately coated panels per formulation (n = 3). The cured coating demonstrated high visible‐light transmittance (89.6 ± 0.6%, Figure 7A and Table S6), a pencil hardness of 2H on all three panels, ISO 2409 Class 0 cross‐cut adhesion, a critical scratch load of 6.6 ± 1.1 N, and impact resistance of 2.94 J during rapid‐deformation testing. Furthermore, under ISO 2812–4 spotting conditions, the coating maintained a Class 0 rating after exposure to a spectrum of harsh agents, including water, hot water, hydrochloric acid, sodium hydroxide, ethanol (50% and 95%), and diesel fuel (Figure 7B), indicating resistance to these reagents under short‐duration laboratory spotting conditions. In contrast, the PM‐3‐ATPE‐Coating control showed comparatively lower values in most of the tests shown in Figure 7, consistent with beneficial structural effects of NPOSS. When applied as a final clear topcoat on the automotive model, PM‐3‐NPOSS exhibited favorable transparency, gloss, and leveling (Figure 7C).

The pot life of the PM‐3/NPOSS formulation was evaluated by monitoring film‐forming performance, fineness of grind, 60° gloss, and pencil hardness over time after mixing (Figure S6 and Table S7 in the SI). The formulation remained processable at least 12 h at room temperature, maintaining continuous film formation, fineness below 5 µm, stable 60° gloss (above 81 GU), and pencil hardness of 2H.

The thermal cycling resistance of the coatings was evaluated by subjecting PM‐3‐NPOSS‐Coating and PM‐3‐ATPE‐Coating to 18 cycles between − 20°C and 60°C (2 h dwell at each temperature, Figure S7). After thermal cycling, PM‐3‐NPOSS‐Coating showed no cracking, blistering, or delamination. The cross‐cut adhesion remained at Class 0 on all three panels, and the pencil hardness was maintained at 2H (Table S8). In contrast, the PM‐3‐ATPE‐Coating control exhibited some loss of adhesion under the same conditions, with the cross‐cut rating decreasing from Class 1 to Class 4.

Collectively, these results provide preliminary laboratory‐scale evidence of properties relevant to protective clearcoat applications.

## 3 Conclusion

In conclusion, we demonstrate a coalescent‐free waterborne thermoset coating strategy wherein a water‐soluble amine‐functionalized POSS appears to simultaneously orchestrate latex particle coalescence and interparticle crosslinking within an epoxy‐functionalized microsphere latex. By transiently plasticizing the latex particles while maintaining sufficiently moderated amine–epoxy reactivity, NPOSS offers a possible route to mitigate the long‐standing conflict between diffusion‐driven film formation and premature network immobilization, enabling continuous film formation without added VOC‐emitting coalescing agents or co‐solvents. The resulting films exhibit a multiscale reinforcement architecture comprising a molecular‐scale covalent network, nanoscale POSS‐associated heterogeneous nanostructures, and micrometer‐scale compositional homogeneity. This architecture gives rise to a combination of strength, toughness, thermal properties, and chemical resistance. These findings demonstrate the potential of NPOSS/latex film as an effective approach for high‐performance coalescent‐free waterborne thermoset coatings.

## 4 Experimental Section

### 4.1 Materials

Methyl methacrylate (MMA, 99%), 2‐ethylhexyl acrylate (2‐EHA, 98%, stabilized with 4‐methoxyphenol), glycidyl methacrylate (GMA, 98%, stabilized with MEHQ), 2,2′‐azobis(2‐methylpropionamidine) dihydrochloride (AIBA, 98%), tetradecyltrimethylammonium bromide (TTAB, 98%), cetyltrimethylammonium bromide (CTAB, 99%), amine‐terminated polypropylene glycol trimethylolpropane ether (ATPE, ZT‐143, average Mn ≈ 440), and (3‐aminopropyl)triethoxysilane (99%) were purchased from Energy‐Chemical unless otherwise stated. ER‐30 was purchased from ADEKA (Japan). Isobornyl methacrylate (IBOMA, 85%) was purchased from Leyan (China). Rectangular aluminum panels (70 mm × 150 mm × 0.5 mm) were obtained from Biuged (Guangzhou, China). Deionized (DI) water was used in all experiments. All reagents were used as received unless otherwise noted.

### 4.2 Synthesis and Characterization of Polymeric Microspheres

#### 4.2.1 Synthesis of Epoxy‐Functionalized Microsphere Latex With Two‐Stage Seeded Core–Shell Architecture

Epoxy‐functionalized polymeric microspheres with intended two‐stage seeded core–shell architecture were synthesized by seeded emulsion polymerization. For PM‐1, pre‐emulsion I was prepared by mixing MMA (21.03 g), 2‐EHA (12.08 g), CTAB (0.27 g), ER‐30 (0.52 g), and DI water (33.5 mL) at 1000 rpm for 3 h. Pre‐emulsion II followed the same procedure, incorporating MMA (18.11 g), 2‐EHA (8.96 g), GMA (6.04 g), CTAB (0.27 g), ER‐30 (0.52 g), and DI water (33.5 mL).

All polymerizations were carried out under argon in a 1000 mL jacketed glass reactor equipped with a mechanical stirrer and a reflux condenser. Initially, CTAB (0.30 g), ER‐30 (0.25 g), and DI water (40.0 mL) were added to the reactor. A portion of pre‐emulsion I (6.00 g, 8.9 wt % of the total) was introduced as the seed feed while stirring at 1000 rpm. This was followed by adding AIBA (0.12 g) dissolved in DI water (3.0 mL). The reactor was then heated to 85°C and maintained for 20 min. Subsequently, the remaining pre‐emulsion I (61.4 g, 91.1 wt %) was fed over 1.5 h using a peristaltic pump. Simultaneously, an aqueous AIBA solution (0.10 g in 5.0 mL DI water) was added with a syringe pump. After the feed was complete, the reaction continued at 85°C for 1 h.

Pre‐emulsion II was added over 1.5 h using the same method, along with a second AIBA solution (0.10 g in 5.0 mL DI water). The reaction mixture was kept at 85°C for another hour, then cooled to 40°C. It was filtered through a 75 µm mesh to remove coagulum. The resulting latex was labeled PM‐1.

PM‐2 and PM‐3 were synthesized following the protocol (Table 2), which details the monomer and surfactant compositions. In the synthesis of PM‐3, IBOMA was added to pre‐emulsion II, and CTAB was substituted with TTAB at the same mass loading. Unless otherwise specified, PM‐1 was used for mechanistic and structural studies, PM‐2 for mechanical testing, and PM‐3 for coating‐performance evaluation. Portions of the latex were lyophilized until a constant mass was achieved.

**TABLE 2:** The basic formula of epoxy‐functionalized microsphere latexes with two‐stage seeded core–shell architecture.

Component	PM‐1 PE‐I^a^ (g)	PM‐1 PE‐II^b^ (g)	PM‐1 BS^c^ (g)	PM‐2 PE‐I (g)	PM‐2 PE‐II (g)	PM‐2 BS (g)	PM‐3 PE‐I (g)	PM‐3 PE‐II (g)	PM‐3 BS (g)
MMA	21.03	18.11	——	7.94	18.11	——	11.06	18.11	——
2‐EHA	12.08	8.96	——	25.45	8.96	——	22.05	8.96	——
GMA	——	6.04	——	——	6.04	——	——	6.04	——
IBOMA	——	——	——	——	——	——	——	6.62	——
CTAB	0.27	0.27	0.30	0.27	0.27	0.30	——	——	——
TTAB	——	——	——	——	——	——	0.27	0.27	0.30
ER‐30	0.52	0.52	0.25	0.52	0.52	0.25	0.52	0.52	0.25

^a^ PE‐I——Pre‐Emulsion‐I;

^b^ PE‐II——Pre‐Emulsion‐II;

^c^ BS——Before Seeded.

#### 4.2.2 Characterization of Polymeric Microspheres with Two‐Stage Seeded Core–Shell Architecture

The hydrodynamic diameter, particle‐size distribution, and zeta potential were measured by dynamic light scattering (DLS) with a Malvern Panalytical Zetasizer Nano ZS90. Each latex was diluted 2000‐fold with DI water before measurement. For size analysis, about 1 mL of the diluted latex was placed in a quartz cuvette and measured at 25°C using the instrument's fixed optical geometry and a 632.8 nm laser. Three measurements were taken for each sample, and the hydrodynamic diameter and polydispersity index (PDI) were determined through cumulant analysis with the Zetasizer software. For zeta potential analysis, the diluted latex was loaded into DTS1070 folded capillary cells after visible bubbles were removed. Electrophoretic mobilities were then converted to zeta potentials using the Smoluchowski approximation.

The molecular‐weight distribution of the polymers was determined using gel permeation chromatography (GPC) on an Agilent 1260 Infinity II instrument, equipped with UV and differential refractive index detectors. A lyophilized polymer sample (5 mg) was dissolved in 1 mL of THF through sonication, and the solution was filtered using a 0.22 µm GVHP membrane. Only the fraction soluble in THF was analyzed. The measurements were conducted on Agilent PLgel 5 µm Mixed‐D columns (300 × 7.5 mm) at 40°C, with THF as the mobile phase flowing at 1.0 mL/min. Polystyrene standards with molecular weights of 650000, 290000, 123000, 12980, 4910, and 3050 g/mol were used for calibration. The number‐average molecular weight (M_n_), weight‐average molecular weight (M_w_), and dispersity (Đ) were calculated using Agilent GPC software.

Transmission electron microscopy (TEM) was conducted using a JEOL JEM‐1200EX microscope. The latex samples were diluted 2500‐fold with deionized water and negatively stained with 1 wt % aqueous phosphotungstic acid before imaging.

Differential scanning calorimetry (DSC) was conducted using a TA Q200 instrument. Each lyophilized polymer sample, weighing approximately 5 mg, was sealed in an aluminum pan. The samples were then heated from −50 to 200°C at a rate of 10°C per min under a nitrogen atmosphere. One specimen was analyzed for each formulation (n = 1). Accordingly, the thermograms and Tg values are reported as individual measurements without error estimates or inferential statistical comparison.

### 4.3 Synthesis and Characterization of Octa(3‐aminopropyl)‐POSS

#### 4.3.1 Synthesis of Octa(3‐aminopropyl)‐POSS

Octa(3‐aminopropyl)‐POSS (NPOSS) was synthesized using a method adapted from the literature [64]. In a typical procedure, (3‐aminopropyl)triethoxysilane (15 mL, 60 mmol) and concentrated hydrochloric acid (37 wt %, 30 mL) were added to methanol (350 mL). The mixture was stirred at 70°C for 18 h. Afterward, the reaction mixture was poured into tetrahydrofuran (90 mL) to precipitate the product. The precipitate was washed three times with tetrahydrofuran (30 mL each time). The solid was collected via vacuum filtration and dried under reduced pressure to eliminate residual solvent, yielding a white NPOSS powder (2.81 g, 30%).

#### 4.3.2 Characterization of Octa(3‐aminopropyl)‐POSS

^1^H and ^13^C{^1^H} NMR spectra were recorded on a Bruker Avance NEO 500 spectrometer. ^29^Si{^1^H} NMR spectra were recorded on a Bruker Avance III spectrometer. Spectra were processed using MestReNova (Mestrelab). Chemical shifts are reported in ppm relative to residual DMSO‐*d*_6_ (δ_H_ = 2.50 ppm and δ_C_ = 39.52 ppm). Signal multiplicities are denoted as s (singlet), d (doublet), t (triplet), q (quartet), and m (multiplet). High‐resolution mass spectra (HRMS) were acquired on a Thermo Scientific Q Exactive Hybrid Quadrupole‐Orbitrap mass spectrometer.

NPOSS was characterized by ^1^H NMR, ^13^C{^1^H} NMR, ^29^Si{^1^H} NMR (Figures S8–S10), with HRMS data provided below. ^1^H NMR (500 MHz, DMSO‐*d*_6_, δ): 8.25 (s, 24H), 2.78 (m, 16H), 1.73 (m, 16H), 0.74 (m, 16H). ^13^C{^1^H} NMR (126 MHz, DMSO‐*d*_6_, δ): 41.00, 20.59, 8.42. ^29^Si{^1^H} NMR (79 MHz, DMSO‐*d*_6_, δ): −66.5. HRMS (m/z, [M+H]^+^): calculated 881.2876; found 881.2883.

To evaluate the extent of amine protonation in as‐isolated NPOSS quantitatively, we determined the chloride content in its aqueous solution. We dissolved NPOSS powder in deionized water to achieve a concentration of 31.60 mg/mL. The chloride content was measured using the silver nitrate potentiometric titration method outlined in GB/T 5750.5‐2023. In this process, an aliquot of the NPOSS solution was titrated with a standardized AgNO_3_ solution under continuous stirring, while the potential was recorded relative to the titrant volume. The endpoint was identified at the inflection point of the potential–volume curve. We calculated the chloride mass concentration, ρ(Cl^−^), following the standard procedure. Assuming the detected chloride primarily originated from –NH3^+^Cl^−^ ion pairs in NPOSS, we estimated the average ammonium degree based on the measured chloride concentration, the NPOSS solution's mass concentration, and the molecular weight of the neutral POSS cage obtained from HRMS (77.4%, the estimated fraction of protonated amine groups, Table S4).

### 4.4 Preparation and Characterization of Crosslinked NPOSS/Latex Films

#### 4.4.1 Preparation of Crosslinked NPOSS/Latex Films

Crosslinked films were prepared by curing the epoxy‐functionalized microspheres with amine‐functional curing agents. NPOSS was dissolved in DI water at 31.60 mg/mL. For comparison, ATPE was dissolved in DI water at 37.15 mg/mL. Each curing‐agent solution was mixed with the latex at an amine‐to‐epoxy equivalent ratio of 2:1. In a typical formulation, 2 mL of the curing‐agent solution was mixed with 1 g of latex. For blank controls, the curing‐agent solution was replaced with an equal volume of DI water to keep the dilution ratio comparable. The nominal amine‐to‐epoxy equivalent ratio was calculated based on the total aminopropyl content of NPOSS, without correcting for the protonated fraction.

Aliquots (0.85 mL) from each formulation were poured into dumbbell‐shaped PTFE molds (50 mm × 10 mm × 1 mm), dried at room temperature for 6 h, and then cured overnight at 60°C for PM‐1‐based formulations, room temperature for PM‐2‐based formulations, and 60°C for PM‐3‐based formulations to ensure sufficient crosslinking for structural characterization. The cured specimens were used for subsequent structural, thermal, and mechanical characterization.

#### 4.4.2 Monitoring of Crosslinking and Morphological Evolution

To monitor curing in aqueous media, NPOSS solution and the microsphere latex were mixed at the same amine‐to‐epoxy equivalent ratio (2:1) used for bulk thermosets. The freshly mixed formulation was used directly for ATR‐FTIR, DLS, and cryo‐TEM measurements.

ATR‐FTIR monitored the curing and drying behavior of a thin aliquot on the ATR crystal. A 20 µL sample of the mixed dispersion was placed on the ATR crystal, with the temperature maintained at 50°C during the measurement. 50°C was the lowest temperature at which the instrument could maintain stable temperature control. This temperature was selected as a compromise to accelerate water evaporation for spectral clarity while maintaining a moderate reaction rate to capture the kinetic profile of the amine–epoxy reaction. Spectra were collected every 10 min for the first 12 h and then every hour thereafter. The study tracked the reaction by observing the evolution of the band at 908 cm^−1^ (epoxide group, C‐O‐C asymmetric stretching vibration) and the band at 1510 cm^−1^ (assigned to amine‐related N–H deformation vibration). Peak intensities were normalized before conducting kinetic analysis.

Particle‐size evolution during curing was analyzed by Dynamic Light Scattering (DLS). Aliquots were collected at predetermined time points up to 280 min for the PM‐1/NPOSS system and up to 720 min for the PM‐1/ATPE control. Each sample was diluted 2000‐fold with deionized water and measured according to the previously described method.

The morphological evolution of the mixed formulation was examined by cryogenic transmission electron microscopy (cryo‐TEM, FEI Talos F200C). Aliquots were taken at 0, 60, 180, and 280 min and diluted 140‐fold with DI water before grid preparation. The 0 and 60‐min samples were placed on holey carbon‐coated copper grids, while the 180 and 280‐min samples were placed on ultrathin carbon‐coated grids. The grids were vitrified by plunge‐freezing in liquid ethane and then transferred to the microscope using a cryogenic holder.

#### 4.4.3 Morphological Characterization of Cured Samples

For analyzing nanoscale and microscale morphology, the cured dumbbell specimens were sectioned, embedded in epoxy resin, and cryo‐polished to achieve flat cross‐sections.

AFM‐IR measurements were carried out on a Bruker NanoIR3‐S instrument in tapping mode using a quantum cascade laser. Chemical maps were recorded at 1510 and 1725 cm^−1^ to evaluate the spatial distribution of the NPOSS‐associated and polymer‐associated regions. The lateral spatial resolution was approximately 10 nm. Detailed procedures for the AFM‐IR intensity distribution and analysis were described in the Supporting Information (Section S4).

Scanning electron microscopy coupled with energy‐dispersive X‐ray spectroscopy (SEM‐EDS) was used to analyze microscale morphology and elemental distributions. The polished cross‐sections were sputter‐coated with a thin Au layer and examined under high vacuum. Elemental maps for Si, O, and C were collected from the same cross‐sections. The spatial resolution of SEM‐EDS mapping was approximately 100 nm.

Small‐angle X‐ray scattering (SAXS) measurements were performed on a Xenocs Xeuss 3.0 instrument in transmission mode. Cross‐sections of the cured specimens were mounted perpendicular to the X‐ray beam, and scattering data were collected over q = 1 – 10 nm−1. The resulting profiles were analyzed to estimate characteristic domain sizes and structural correlations in the cured network.

#### 4.4.4 Thermal and Mechanical Characterization

DSC was conducted using a TA Q200 instrument. Each cured specimen, weighing approximately 5 mg, was sealed in an aluminum pan. The samples were then heated from −50 to 200°C at a rate of 10°C per min under a nitrogen atmosphere. One specimen was analyzed for each formulation (n = 1). Accordingly, the thermograms and Tg values were reported as individual measurements without error estimates or inferential statistical comparison.

Uniaxial tensile tests were conducted at 25 ± 2°C using a Shimadzu AGS‐X‐50N universal testing machine with a crosshead speed of 10 mm/min. Three specimens were tested for each formulation (n = 3). Tensile strength, elongation at break, and elastic modulus were reported as mean ± SD.

#### 4.4.5 Gel Content

Gel content was determined by Soxhlet extraction. Each cured bulk sample, weighing approximately 2 g, was wrapped in quartz‐fiber filter paper and extracted with 150 mL of ethyl acetate at 85°C for 12 h. Gel content measurements were performed using three separately weighed specimens for each formulation (n = 3), and the results were reported as mean ± SD. The residue was then dried under vacuum until it reaches a constant mass. Gel content (G) was calculated using Equation (3), based on the ratio of the residual mass to the initial mass.

(3) $$G\%=\frac{m_{r}}{m_{r0}}\times 100\%$$

where: m_r_ ‐ residual mass of the sample, g; and m_r0_ ‐ original mass of the sample, g.

### 4.5 Preparation and Performance Evaluation of Coatings

#### 4.5.1 Preparation of Coating Formulations and Films

NPOSS and ATPE stock solutions were prepared as detailed in Section 4.4.1 and mixed separately with the microsphere latex at an amine‐to‐epoxy equivalent ratio of 2:1. These formulations, without added co‐solvent or coalescing agents, were spray‐coated onto pre‐cleaned aluminum panels (70 mm × 150 mm × 0.5 mm) and glass panels (29.7 mm × 42.0 mm × 1.0 mm). The coated substrates were cured at 120°C for 6 h and then left at room temperature for 3 days.

#### 4.5.2 Coating Performance Tests

The dry‐film thickness of coated aluminum panels was measured using a magnetic‐induction thickness gauge, following ASTM D7091‐13 standards. Three separately coated panels were evaluated for each formulation (n = 3). Five positions were measured on each panel and averaged to obtain one panel‐level thickness. The reported value represented the mean ± SD of the three panel‐level averages.

Pencil hardness was assessed following ISO 15184: 2020 using CHUNG HWA pencils and a standard pencil‐hardness tester. The highest pencil grade that did not create a continuous scratch exceeding 3 mm was noted. Three separately coated panels were tested for each formulation (n = 3), and the panel‐level grades were reported. Pencil hardness was reported as the consistent rating obtained from three parallel measurements; the lowest rating was reported when inconsistencies occurred.

Cross‐cut adhesion was assessed following ISO 2409: 2020 using a BGD503 multi‐blade cutter. For the coating thicknesses used here, a blade spacing of 1 mm was used. Adhesive tape (75 mm wide) was applied over the lattice, removed rapidly, and the adhesion rating was assigned according to the ISO scale. Cross‐cut adhesion was evaluated on three separately coated panels per formulation (n = 3). Because the ISO adhesion class was an ordinal result, the panel‐level classifications were reported as the consistent rating obtained from three parallel measurements; the lowest rating was reported when inconsistencies occurred.

Scratch resistance was assessed following ISO 1518‐2: 2011 using a BGD 5202 motorized scratch tester equipped with a 1.0 mm hemispherical tungsten‐carbide stylus. The normal load was increased linearly from 0.5 to 10.0 N along the scratch path. The failure track was inspected under an optical microscope (40 ×), and the critical load (F_c_) was determined from the position of the first visible coating failure. Three separately coated panels were tested for each formulation (n = 3), and the critical scratch loads were reported as mean ± SD.

Impact resistance was assessed following ISO 6272‐2:2011 standards. A BGD 301 impact tester equipped with a hemispherical impactor (12.7 ± 0.1 mm) and a 1000 g falling weight was used. The maximum impact energy that did not result in visible cracking, wrinkling, or delamination was determined using Equation (4). Impact resistance was evaluated on three separately coated panels per formulation (n = 3). The reported value represented the maximum impact energy passed by all three panels.

(4) E=m×g×h

where: *E* – the impact energy in joules (J); *m* – the mass of the falling weight in kilograms (kg); *g* – the acceleration due to gravity (9.81 m / s^2^); *h* – the drop height in meters (m)

Optical transmittance was measured on coated glass panels using an LS183 transmittance meter (Linshang, China), which simultaneously recorded UV transmittance at a peak wavelength of 365 nm, IR transmittance at a peak wavelength of 940 nm, and visible‐light transmittance over the 380–760 nm range according to the CIE photopic luminosity function. PM‐3‐NPOSS and PM‐3‐ATPE formulations were spray‐coated onto pre‐cleaned glass panels (29.7 mm × 42.0 mm × 1.0 mm), cured at 120°C for 6 h, and conditioned at room temperature for 3 days before measurement. The corresponding dry‐film thicknesses, determined using identically prepared aluminum witness panels, were 11.5 ± 0.6 µm for PM‐3‐NPOSS and 11.7 ± 2.7 µm for PM‐3‐ATPE. Three separately coated glass panels were evaluated for each formulation (n = 3); three positions were measured on each panel and averaged to obtain one panel‐level value, and the results were reported as mean ± SD across the three panels. Uncoated glass panels of the same dimensions were measured separately as substrate controls.

Chemical resistance was evaluated by spot testing according to ISO 2812–4:2017. The reagents used included distilled water, hot water (boiling water added initially and allowed to cool during the test), ethanol (95% and 50%), HCl (10 wt % in DI water), NaOH (5 wt % in DI water), and diesel fuel. For each test, 0.1 mL of the reagent was placed on the coated surface and covered with a glass Petri dish. After 1 h exposure, the reagent was removed using a lint‐free cloth. Samples were rinsed with DI water and dried. The coatings were then allowed to recover at room temperature for 24 h before visual rating. The ratings shown in Figure 7B correspond to 1 h exposure followed by 24 h recovery. For each reagent and formulation, three separately coated panels were tested (n = 3). Because the resulting ISO classifications are ordinal data, the panel‐level classifications were reported without numerical averaging. Where all three panels showed the same classification, the common classification is reported; the lowest rating was reported when inconsistencies occurred.

#### 4.5.3 Laboratory‐Scale Coating Demonstration for High‐Performance Topcoat Applications

For a laboratory‐scale demonstration on complex surfaces, the coating formulation described in Section 4.5.1 was spray‐applied to primer‐coated aluminum automotive model substrates (196 mm × 100 mm × 50 mm). After application, the coated models were cured at 120°C for 6 h, and were left at room temperature for 3 days. The models were visually inspected for coating uniformity, surface smoothness, and apparent adhesion.

### 4.6 Statistical Analysis

Unless otherwise stated, continuous quantitative data were presented as mean ± standard deviation (SD, the statistical meaning of the error bars). The value of n refers to the number of tested specimens or separately coated panels rather than repeated instrumental readings collected from the same specimen. Measurements obtained at multiple positions on a single panel were treated as technical replicates and averaged to generate one panel‐level value.

## Author Contributions

**Jie An**: conceptualization, writing – original draft, writing – review and editing, data curation, project administration. **Chao Wang**: conceptualization, methodology, data curation, investigation, visualization, writing – original draft, project administration. **Ruoyan Yang**: data curation, supervision. **Bin Wu**: validation, methodology. **Lin Li**: methodology, validation. **Minlong Wang**: conceptualization, data curation, supervision, formal analysis. **Yibo Ru**: validation. **Xiaohe Zhang**: supervision, data curation.

## Funding

This project was supported by Chinese Universities Scientific Fund (2025TC119) and Beijing Qi Dian Shi Neng Technology Co., Ltd.

## Conflicts of Interest

The authors declare no conflicts of interest.

## Supporting information




**Supporting File**: advs77495‐sup‐0001‐SuppMat.pdf.

## Data Availability

Data supporting the findings of this study are available from the corresponding author upon reasonable request.

## References

[1] M. U. Ali , M. Z. Gulzar , B. Sattar , et al., “Silent Threats of Lead‐Based Paints In Toys and Households to Children's Health and Development,” Journal of Hazardous Materials 486 (2025): 136984. 39740545 10.1016/j.jhazmat.2024.136984

[2] L. Pilon , D. Day , H. Maslen , et al., “Development of a solvent Sustainability Guide for the Paints and Coatings Industry,” Green Chemistry 26 (2024): 9697–9711.

[3] M. Qin , B. N. Murphy , K. K. Isaacs , et al., “Criteria Pollutant Impacts of Volatile Chemical Products Informed by Near‐Field Modelling,” Nature Sustainability 4 (2021): 129–137. 10.1038/s41893-020-00614-1 PMC7592713 33134558

[4] C. E. Stockwell , M. M. Coggon , G. I. Gkatzelis , et al., “Volatile Organic Compound Emissions From Solvent‐ and Water‐Borne Coatings – Compositional Differences and Tracer Compound Identifications,” Atmospheric Chemistry and Physics 21 (2021): 6005–6022.

[5] K. Pieters and T. H. Mekonnen , “Progress in Waterborne Polymer Dispersions for Coating Applications: Commercialized Systems and New trends,” RSC Sustainability 2 (2024): 3704–3729.

[6] A. Arjmandi , H. Bi , S. U. Nielsen , and K. Dam‐Johansen , “From Wet to Protective: Film Formation in Waterborne Coatings,” ACS Applied Materials & Interfaces 16 (2024): 58006–58028. 39431441 10.1021/acsami.4c09729 PMC11533165

[7] J. L. Keddie , “Film Formation of Latex,” Materials Science and Engineering: R: Reports 21 (1997): 101–170.

[8] P. A. Steward , J. Hearn , and M. C. Wilkinson , “An Overview of Polymer Latex Film Formation and Properties,” Advances in Colloid and Interface Science 86 (2000): 195–267. 10997764 10.1016/s0001-8686(99)00037-8

[9] Y. Wang and M. A. Winnik , “Polymer Diffusion Across Interfaces in Latex Films,” The Journal of Physical Chemistry 97 (1993): 2507–2515.

[10] S. M. Dron , S. J. Bohorquez , D. Mestach , and M. Paulis , “Reducing the Amount of Coalescing Aid in High Performance Waterborne Polymeric Coatings,” European Polymer Journal 170 (2022): 111175.

[11] J. Kaur , R. Krishnan , B. Ramalingam , and S. Jana , “Hydroxyethyl Sulfone Based Reactive Coalescing Agents for low‐VOC Waterborne Coatings,” RSC Advances 10 (2020): 17171–17179. 35521475 10.1039/d0ra00753f PMC9053402

[12] E. M. Dogan‐Guner , S. Brownell , G. T. Schueneman , M. L. Shofner , and J. C. Meredith , “Enabling Zero Added‐Coalescent Waterborne Acrylic Coatings With Cellulose Nanocrystals,” Progress in Organic Coatings 150 (2021): 105969.

[13] H. Kang , S. Aswale , H. Cho , et al., “Development of Eco‐Friendly Waterborne Polyurethane 2 K Clearcoats Through Reactive Surfactants and Internal Particle Crosslinking,” Progress in Organic Coatings 208 (2025): 109500.

[14] R. Krishnan , J. Kaur , B. Ramalingam , and S. Jana , “Amine Functional Reactive Coalescing Agents (RCA) for Emission‐Free Waterborne Paints And Coatings,” Chemical Communications 60 (2024): 11331–11334. 39297804 10.1039/d4cc03147d

[15] J. Feng , H. Pham , P. Macdonald , et al., “Formation and Crosslinking of Latex Films Through the Reaction of Acetoacetoxy Groups With Diamines Under Ambient Conditions,” Journal of Coatings Technology 70 (1998): 57–68.

[16] S. Tariq , L. Irusta , M. Fernández , and M. Paulis , “Kinetic Study of Crosslinking Between Acetoacetoxy and Hexamethylene Diamine Functionalized Waterborne Latexes in Two‐Pack Systems,” Progress in Organic Coatings 165 (2022): 106732.

[17] M. A. Winnik , “Interdiffusion and Crosslinking in Thermoset Latex Films,” Journal of Coatings Technology 74 (2002): 49–63.

[18] T. Tamai , P. Pinenq , and M. A. Winnik , “Effect of Cross‐Linking on Polymer Diffusion in Poly(butyl methacrylate‐ co‐Butyl acrylate) Latex Films,” Macromolecules 32 (1999): 6102–6110.

[19] M. A. Winnik , P. Pinenq , C. Krüger , J. Zhang , and P. V. Yaneff , “Crosslinking vs. interdiffusion Rates in Melamine‐Formaldehyde Cured Latex Coatings: A Model for Waterborne Automotive Basecoat,” Journal of Coatings Technology 71 (1999): 47–60.

[20] J. W. Taylor and M. A. Winnik , “Functional Latex and Thermoset Latex Films,” JCT Research 1 (2004): 163–190.

[21] R. Liu , M. A. Winnik , F. Di Stefano , and J. Vanketessan , “Interdiffusion vs Cross‐Linking Rates in Isobutoxyacrylamide‐Containing Latex Coatings,” Macromolecules 34 (2001): 7306–7314.

[22] Y. Liu , H. Zhou , K. Tran , et al., “Monitoring Polymer Diffusion in a Waterborne 2K Polyurethane Formulation Based on an Acrylic Polyol Latex,” Macromolecules 53 (2020): 10744–10753.

[23] K. Tran , Y. Liu , M. Soleimani , F. Lucas , and M. A. Winnik , “Waterborne 2‐Component Polyurethane Coatings Based on Acrylic Polyols with Secondary Alcohols,” Progress in Organic Coatings 190 (2024): 108374.

[24] Y. Liu , K. Tran , K. Ho , et al., “Stratification of Polyisocyanate in Two‐Component Waterborne Polyurethane Films,” Chemical Engineering Journal 483 (2024): 148981.

[25] M. Salzano de Luna , “Recent Trends in Waterborne and Bio‐Based Polyurethane Coatings for Corrosion Protection,” Advanced Materials Interfaces 9 (2022): 2101775, 10.1002/admi.202101775.

[26] H. Zhou , Y. Liu , M. Zhang , et al., “Characterization of an Aqueous Dispersion of a Hydrophilic Polyisocyanate for Waterborne Two‐Pack Polyurethane Coatings,” ACS Applied Polymer Materials 2 (2020): 1491–1499.

[27] X. Zhang , Y. Liu , H. Huang , Y. Li , and H. Chen , “The Diacetone Acrylamide Crosslinking Reaction and Its Control of Core‐Shell Polyacrylate Latices at Ambient Temperature,” Journal of Applied Polymer Science 123 (2012): 1822–1832.

[28] C. G. Koukiotis , M. M. Karabela , and I. D. Sideridou , “Mechanical Properties of Films of Latexes Based on Copolymers BA/MMA/DAAM and BA/MMA/VEOVA‐10/DAAM and the Corresponding Self‐Crosslinked Copolymers Using the Adipic acid Dihydrazide as Crosslinking Agent,” Progress in Organic Coatings 75 (2012): 106–115.

[29] N. Kessel , D. R. Illsley , and J. L. Keddie , “The Diacetone Acrylamide Crosslinking Reaction and its Influence on the Film Formation of an Acrylic Latex,” Journal of Coatings Technology and Research 5 (2008): 285–297.

[30] Y. Nakayama , “Development of Novel Aqueous Coatings Which Meet the Requirements of Ecology‐Conscious Society: Novel Cross‐Linking System Based on the Carbonyl–Hydrazide Reaction and Its Applications,” Progress in Organic Coatings 51 (2004): 280–299.

[31] X. Zhu , Q. Zhang , L. Liu , X. Z. Kong , and S. Feng , “Synthesis and Characterization of a New Compound Bearing Ketone and Hydroxyl Groups for Preparation of Ambient Temperature Self‐Crosslinking Waterborne Polyurethanes,” Progress in Organic Coatings 59 (2007): 324–330.

[32] E. M. Eaves and P. A. Lovell , “A Soft–Soft Nanocomposite Approach for Design of Water‐Borne Acrylic Surface Coatings,” Macromolecular Reaction Engineering 19 (2025): 2400032, 10.1002/mren.202400032.

[33] X. Lin , H.‐T. Li , M.‐X. Nie , et al., “Engineering the Properties of Transparent Hybrid Coating toward High Hardness, Excellent Flexibility, and Multifunction,” ACS Applied Materials & Interfaces 14 (2022): 39432–39440. 35993524 10.1021/acsami.2c13256

[34] S.‐W. Kuo and F.‐C. Chang , “POSS related polymer nanocomposites,” Progress in Polymer Science 36 (2011): 1649–1696.

[35] S. Y. Soong , R. E. Cohen , and M. C. Boyce , “Polyhedral Oligomeric Silsesquioxane as a Novel Plasticizer for Poly(vinyl chloride),” Polymer 48 (2007): 1410–1418.

[36] K. N. Raftopoulos and K. Pielichowski , “Segmental Dynamics in Hybrid Polymer/POSS Nanomaterials,” Progress in Polymer Science 52 (2016): 136–187.

[37] C. Wang , X. Zhang , X. Qin , et al., “Critical Influence of Alkene Substitutions on POSS in Enhancing Properties of Polyacrylate Composites,” Materials Today Communications 43 (2025): 111707.

[38] Z. Zhang , Q. Xie , G. Zhang , C. Ma , and G. Zhang , “Multifunctional Recyclable Glassy Polymeric Materials,” Advanced Functional Materials 34 (2024): 2408748, 10.1002/adfm.202408748.

[39] G. Kibar , “Effect of Crosslinking Agent on Mesoporous Spherical POSS Hybrid Particles: Synthesis, Characterization and Thermal Stability,” Journal of Inorganic and Organometallic Polymers and Materials 33 (2023): 831–840.

[40] G. Kibar , “Epoxy Functional Porous POSS Microparticle Synthesis,” Hacettepe Journal of Biology and Chemistry 50 (2022): 359–366.

[41] J. Zhou , X. Zhang , S. Zhao , et al., “Study on the Effects of Soluble POSS on chain Disentanglement in UHMWPE Polymerization,” Polymer 244 (2022): 124561.

[42] A. Romo‐Uribe , “POSS Increased the Toughness and Mechanical Modulus of Acrylic Copolymers by Increasing the Entanglement Density,” Polymer 275 (2023): 125933.

[43] H. Chi , G. Zhang , N. Wang , et al., “Enhancing the Mechanical Strength and Toughness of Epoxy Resins With Linear POSS Nano‐Modifiers,” Nanoscale Advances 4 (2022): 1151–1157. 36131759 10.1039/d1na00757b PMC9419030

[44] K. Imai and Y. Kaneko , “Preparation of Ammonium‐Functionalized Polyhedral Oligomeric Silsesquioxanes with High Proportions of Cagelike Decamer and Their Facile Separation,” Inorganic Chemistry 56 (2017): 4133–4140. 28300405 10.1021/acs.inorgchem.7b00131

[45] C. Wang , B. Zhao , X. Zhang , et al., “Biocide‐Encapsulated Core–Shell Polymer Microspheres: A Novel Approach for Preparing Long‐Lasting Antimicrobial Coatings,” Progress in Organic Coatings 196 (2024): 108722.

[46] N. Jiménez , N. Ballard , and J. M. Asua , “Hard Coatings from Soft Latexes: A Review of Routes to Overcome the Film Formation Dilemma,” Macromolecular Materials and Engineering 309 (2024): 2400026.

[47] C. Cirillo , M. Iuliano , C. Florio , et al., “High Performance Leathers Finishing Through Zero Waste and Metal‐Free Leather Wastes Valorization,” Scientific Reports 15 (2025): 18105. 40413274 10.1038/s41598-025-03182-6 PMC12103561

[48] A. Srivastava , S. Maity , and B. R. Das , “A Review on Multi‐Functional Polyurethane (PU) Coatings for Fabric Applications: Materials, Processes and Recent Developments,” Progress in Organic Coatings 207 (2025): 109377.

[49] Y. Li , L. Zhang , and C. Li , “Highly Transparent and Scratch Resistant Polysiloxane Coatings Containing Silica Nanoparticles,” Journal of Colloid and Interface Science 559 (2020): 273–281. 31634671 10.1016/j.jcis.2019.09.031

[50] J. Lange , A. Luisier , and A. Hult , “Influence of Crosslink Density, Glass Transition Temperature and Addition of Pigment and Wax on the Scratch Resistance of an Epoxy Coating,” Journal of Coatings Technology 69 (1997): 77–82.

[51] F. Obermeier , D. Hense , P. N. Stockmann , and O. I. Strube , “Syntheses and Polymerization of Monoterpene‐Based (meth)acrylates: IBO(M)A as a relevant Monomer for Industrial Applications,” Green Chemistry 26 (2024): 4387–4416.

[52] K. Beshah , A. Izmitli , A. K. Van Dyk , J. J. Rabasco , J. Bohling , and S. J. Fitzwater , “Diffusion‐Weighted PFGNMR Study of Molecular Level Interactions of Loops and Direct Bridges of HEURs on Latex Particles,” Macromolecules 46 (2013): 2216–2227.

[53] S. Wang and R. G. Larson , “Multiple Relaxation Modes in Suspensions of Colloidal Particles Bridged By Telechelic Polymers,” Journal of Rheology 62 (2018): 477–490.

[54] E. Hajizadeh , S. Yu , S. Wang , and R. G. Larson , “A Novel Hybrid Population Balance—Brownian Dynamics Method for Simulating the Dynamics of Polymer‐Bridged Colloidal Latex Particle Suspensions,” Journal of Rheology 62 (2018): 235–247.

[55] L. Zhao , J. Guan , H. Ma , and Z. Sun , “Mechanical Properties and Curing Kinetics of Epoxy Resins Cured by Various Amino‐Terminated Polyethers,” Chinese Journal of Polymer Science 28 (2010): 961–969.

[56] A. C. V. D. dos Santos , B. Lendl , and G. Ramer , “Systematic Analysis and Nanoscale Chemical Imaging of Polymers Using Photothermal‐Induced Resonance (AFM‐IR) Infrared Spectroscopy,” Polymer Testing 106 (2022): 107443.

[57] S. Morsch , Y. Liu , S. B. Lyon , and S. R. Gibbon , “Insights into Epoxy Network Nanostructural Heterogeneity Using AFM‐IR,” ACS Applied Materials & Interfaces 8 (2016): 959–966. 26694687 10.1021/acsami.5b10767

[58] S. Morsch , Y. Liu , P. Greensmith , S. B. Lyon , and S. R. Gibbon , “Molecularly controlled Epoxy Network Nanostructures,” Polymer 108 (2017): 146–153.

[59] C. M. Sahagun , K. M. Knauer , and S. E. Morgan , “Molecular Network Development and Evolution of Nanoscale Morphology in an Epoxy‐Amine Thermoset Polymer,” Journal of Applied Polymer Science 126 (2012): 1394–1405.

[60] K. Mishra , G. Pandey , and R. P. Singh , “Enhancing the Mechanical Properties of an Epoxy Resin Using Polyhedral Oligomeric Silsesquioxane (POSS) as Nano‐Reinforcement,” Polymer Testing 62 (2017): 210–218.

[61] A. Strachota , P. Whelan , J. Kříž , et al., “Formation of Nanostructured Epoxy Networks Containing Polyhedral Oligomeric Silsesquioxane (POSS) Blocks,” Polymer 48 (2007): 3041–3058.

[62] L. Feng , B. N. Benhamida , C.‐Y. Lu , et al., “Fundamentals and Characterizations of Scratch Resistance on Automotive Clearcoats,” Progress in Organic Coatings 125 (2018): 339–347. PMC7539636 33033422

[63] U.S. Environmental Protection Agency (EPA) , ENVIRONMENTAL PROTECTION AGENCY 40 CFR Part 60 Review of Standards of Performance for Automobile and Light Duty Truck Surface Coating Operations, Federal Register 2023, 88, 1991.

[64] Y.‐J. Pu , H. Yuan , M. Yang , B. He , and Z.‐W. Gu , “Synthesis of Peptide Dendrimers With Polyhedral Oligomeric Silsesquioxane Cores via Click Chemistry,” Chinese Chemical Letters 24 (2013): 917–920.

