# Supplementary material for: Water‐Soluble POSS Promotes Concurrent Coalescence and Crosslinking for Multiscale‐Reinforced, Coalescent‐Free Waterborne Coatings
Source: Adv Sci (Weinh). 2026 Aug 29:e77495. Online ahead of print. doi: 10.1002/advs.77495 (PMC13525702; doi:10.1002/advs.77495)
Supplement: Supplementary file 1 [file ADVS-9999-e77495-s001.pdf]

Supporting Information

Water-Soluble POSS Promotes Concurrent Coalescence and Crosslinking for Multiscale-Reinforced, Coalescent-Free Waterborne Coatings

*Chao Wang [1], Bin Wu [1], Lin Li [1], Yibo Ru [1], Xiaohe Zhang [1], Minlong Wang [1], Ruoyan Yang [1] and Jie An [1]**

[1]*Department of Nutrition and Health, China Agricultural University, No. 2 Yuanmingyuan West Road, Beijing 100193, China*

* Corresponding author's E-mail: jie_an@cau.edu.cn

Contents

S1. Supplementary Characterization of Polymeric Microspheres with Two-Stage Seeded Core–shell Architecture 2
S2. Supplementary DSC Characterization of Latex Solids and Corresponding NPOSS-Cured Films 3
S3. Room-temperature Storage Stability of PM-3 Latex 4
S4. Statistical Analysis of AFM-IR Intensity Distributions 5
S5. SAXS Data Analysis and Fitting Procedures 6
S6. Estimated Protonation State of NPOSS 8
S7. Supplementary Characterization Statistics of PM-3-Latex Coatings 9
S8. Pot Life Measurement of the PM-3/NPOSS Formulation 10
S9. Thermal Cycling Test of Coatings 12
S10. NMR Spectra of NPOSS 13
S11. AFM-IR Detection Area for Cross-Section of PM-1-NPOSS-Film 15
S12. Supplementary Statistics of SEM-EDS for PM-1-NPOSS-Film 16

S1. Supplementary Characterization of Polymeric Microspheres with Two-Stage Seeded Core–shell Architecture

The solid content (S) was calculated gravimetrically using **Equation (S1)**:

$$S\ \% = \frac{m_f}{m} \times 100\% \quad \text{(S1)}$$

Where: m_f - the weight of the freeze-dried powder, g; m - the weight of the latex of polymeric microspheres with two-stage seeded core–shell architecture, g.

The monomer conversion (Con) was calculated using **Equation (S2)**:

$$Con\ \% = \frac{S \times m_d - m_e + m_g}{m_m} \times 100\% \quad \text{(S2)}$$

Where: S - the solid content of the aqueous dispersion of core–shell polymeric microspheres, %; m_d - the weight of the aqueous dispersion of polymeric microspheres, g; m_e - the weight of the surfactants, g; m_g - the weight of the dry gel after reaction, g; m_m – the weight of the monomer in reaction, g.

Table S1. Supplementary characterization of the polymeric microspheres with two-stage seeded core–shell architecture.

Entry	Solid content [a] / %	Monomer conversion [a] / %	Zeta potential [a] / mV	Gel Content / %
PM-1	37.7	96.5	44.1	25.0 ± 8.9 [b]
PM-2	37.9	97.0	40.5	27.4 ± 4.9
PM-3	38.0	97.1	34.5	26.1 ± 7.4

[a] The solid content, monomer conversion, and zeta potential are reported as single measurements from the corresponding latex batches. [b] The gel content of the freeze-dried latex powders was determined by Soxhlet extraction with THF for 12 h and is reported as mean ± standard deviation (SD) from three independent measurements (n = 3).

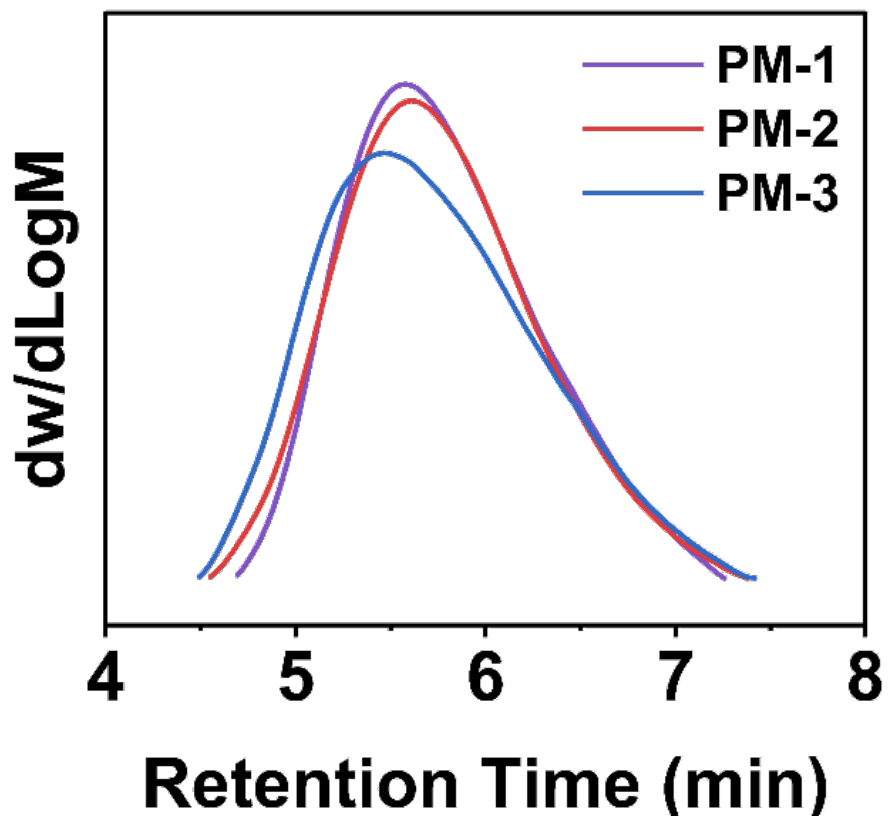

Figure S1. GPC traces of PM-1, PM-2, and PM-3 latex polymers (THF-soluble fraction). The molecular-weight distributions of the THF-soluble fractions of freeze-dried PM-1, PM-2, and PM-3 latex particles were determined by gel permeation chromatography (GPC). The x-axis represents elution time (min), and the y-axis is plotted as dw/d (log M) (weight fraction per log molecular weight interval). The area under each elution peak is normalized to 1.0, allowing direct visual comparison of the molecular weight dispersity across different samples.

S2. Supplementary DSC Characterization of Latex Solids and Corresponding NPOSS-Cured Films

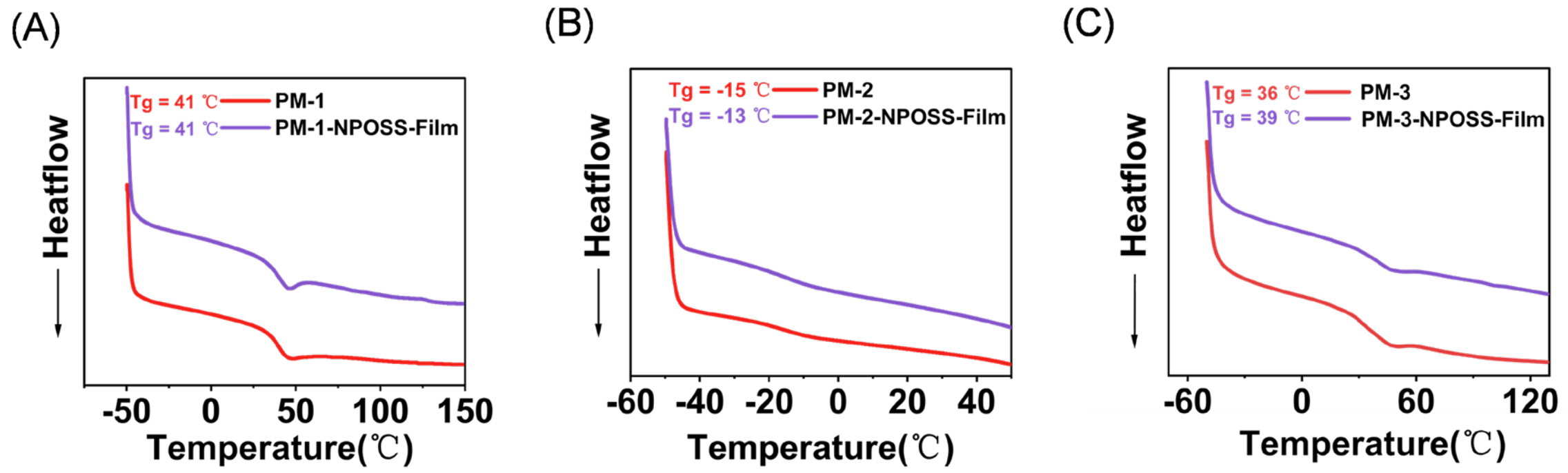

Figure S2. DSC curves of lyophilized polymer sample of PM latex and corresponding NPOSS-crosslinked films.
(A) PM-1 (Tg = 41 °C) and PM-1-NPOSS-Film (Tg = 41 °C).
(B) PM-2 (Tg = -15 °C) and PM-2-NPOSS-Film (Tg = -13 °C).
(C) PM-3 (Tg = 36 °C) and PM-3-NPOSS-Film (Tg = 39 °C).
DSC thermograms were recorded at a heating rate of 10 °C/min under nitrogen atmosphere. The Tg values are annotated in each subplot.

S3. Room-temperature Storage Stability of PM-3 Latex

To evaluate the long-term storage stability of the epoxy latex, a portion of the PM-3 latex was sealed in a glass vial and stored at room temperature (approximately 25 °C) for one year. After this period, the particle size, polydispersity index (PDI), and zeta potential were re-measured by dynamic light scattering (DLS) and electrophoretic light scattering, respectively, and the results were compared with those obtained for the freshly prepared latex. In addition, Fourier transform infrared (FT-IR) spectroscopy was performed on dried films cast from the latex before and after storage to monitor the retention of epoxy functionality. As summarized in Table S2, after one year of storage at room temperature, the PM-3 latex displayed no appreciable changes in particle size, PDI, zeta potential, or the normalized epoxy peak area, indicating good colloidal and chemical stability under conventional storage conditions.

Table S2. Colloidal and spectroscopic characterization of PM-3 latex before and after storage for one year.

Entry	Sample	Size / d. nm	PDI	Zeta Potential / mV
1	PM-3	98	0.052	35
2	PM-3-After-1-Year [a)]	99	0.064	31

[a)] PM-3-After-1-Year means the PM-3 latex which was storaged at room temperature for one year.

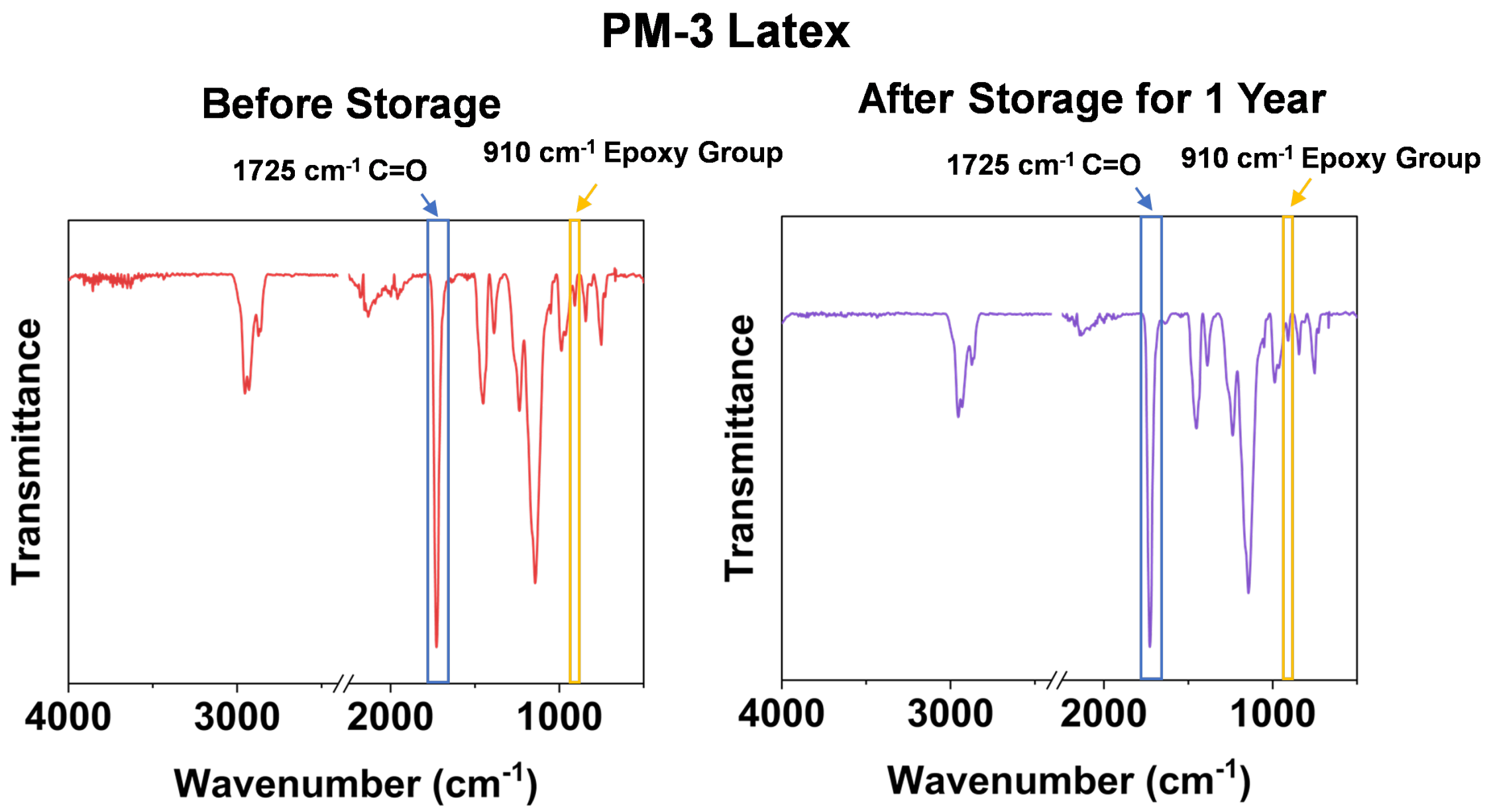

Figure S3. FTIR spectra of PM-3 latex films before (left) and after (right) storage for one year. The blue and yellow vertical lines indicate the ester C=O stretching band (1725 cm^{-1}) and the epoxy C–O–C stretching band (910 cm^{-1}).

S4. Statistical Analysis of AFM-IR Intensity Distributions

The AFM-IR maps acquired at 1725 and 1510 cm^{-1} were exported as two-dimensional pixel-intensity matrices from the same scanned region. Export-border pixels were removed, and only the common valid pixel region was used for analysis. Each map was normalized to its median intensity:

$$I_{\text{norm}}(x,y) = \frac{I(x,y)}{I_M}$$

where I(x,y) is the intensity of a given pixel and I_M is the median intensity of all valid pixels in the corresponding map.

Pixel-intensity histograms were generated from the normalized intensity values. The probability density for each bin was calculated as:

$$P_i = \frac{n_i}{N\Delta I}$$

where n_i is the number of pixels in bin i, N is the total number of valid pixels, and ΔI is the bin width. Thus, the total integrated area of the histogram equals 1.

The coefficient of variation was calculated as:

$$CV = \frac{\sigma}{\mu}$$

where σ and μ are the standard deviation and mean of the normalized pixel-intensity distribution, respectively.

To compare the local amine-associated signal with the acrylic-matrix reference signal, a pixel-wise normalized ratio was calculated:

$$R(x,y) = \frac{I_{1510}(x,y)/I_{M1510}}{I_{1725}(x,y)/I_{M1725}}$$

The median value and percentile range of R were used to describe the distribution of local relative signal intensity.

S5. SAXS Data Analysis and Fitting Procedures

The raw SAXS data were background-subtracted using the scattering of the empty cell. Two complementary models were employed for structural interpretation.

Guinier Analysis:

In the very low-q region where $q \times R_g \leq 1.3$, the scattering intensity follows the Guinier approximation:

$$\ln[I(q)] = \ln(I_0) - \frac{R_g^2}{3} q^2$$

where Rg is the radius of gyration and I_0 is the zero-angle intensity.

Debye-Bueche (D-B) Analysis:

For a statistically isotropic two-phase system, the D-B model correlates the scattering intensity with the correlation function:

$$I(q) = \frac{I_0}{(1 + q^2 \xi^2)^2} + I_{bkg}$$

where ξ is the correlation length (a measure of the average size of homogeneous regions), and I_{bkg} is the background intensity. Since the background was subtracted during preprocessing, I_{bkg} was fixed to zero during the non-linear least-squares fitting. The fitting range was set $q \leq 0.015$ Å^{-1}, for which $q_{max} \cdot \xi$ values are approximately 1.3 – 1.4, slightly exceeding the strict theoretical limit but still within the quasi-linear regime, justifying the use of this model as a reliable phenomenological descriptor.

All non-linear fittings were performed using the Levenberg-Marquardt algorithm in Origin. The extracted parameters are summarized in Table S3. The corresponding Guinier plots and D-B fitting curves for samples PM-1, PM-2, and PM-3 are displayed in Figure S5, respectively.

Table S3. Summary of SAXS fitting parameters for PM-1, PM-2, and PM-3.

Sample	Guinier Rg [a] / nm	D-B ξ [b]/ nm
PM-1-NPOSS-Film	13.1	8.88
PM-2-NPOSS-Film	16.0	9.70
PM-3-NPOSS-Film	22.3	9.08

[a] Rg = radius of gyration obtained from Guinier analysis; [b] ξ = correlation length from Debye–Bueche (D-B) fitting

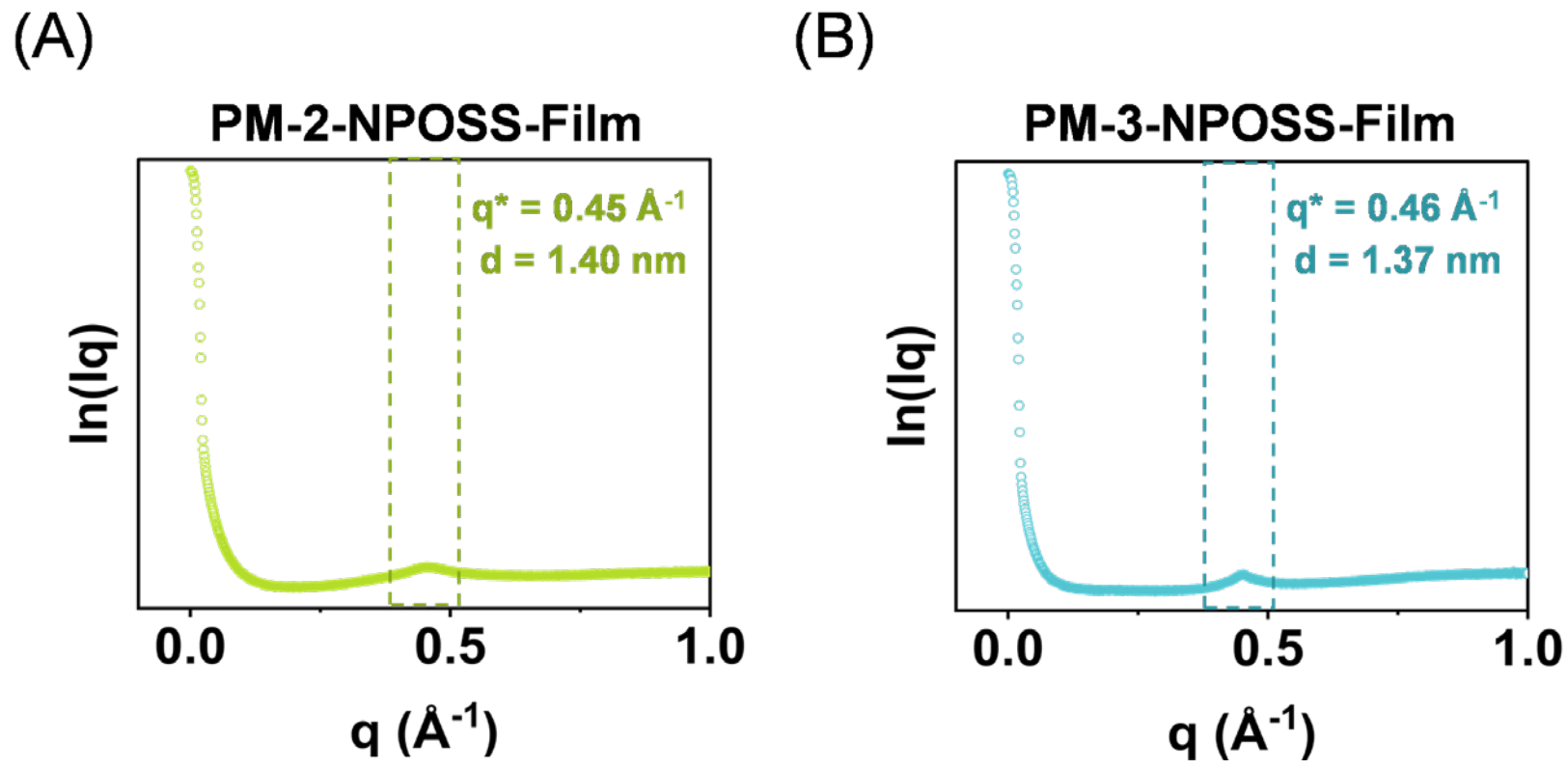

Figure S4. SAXS curve of PM-2-NPOSS-Film and PM-3-NPOSS-Film.
The weak, broad scattering features observed at q* = 0.45 $Å^{-1}$ (d = 1.40 nm) for PM-2 and q* = 0.46 $Å^{-1}$ (d = 1.37 nm) for PM-3 are consistent with short-range structural correlations rather than long-range ordered nanostructures, similar to the feature observed for PM-1-NPOSS-Film (Figure 5A).

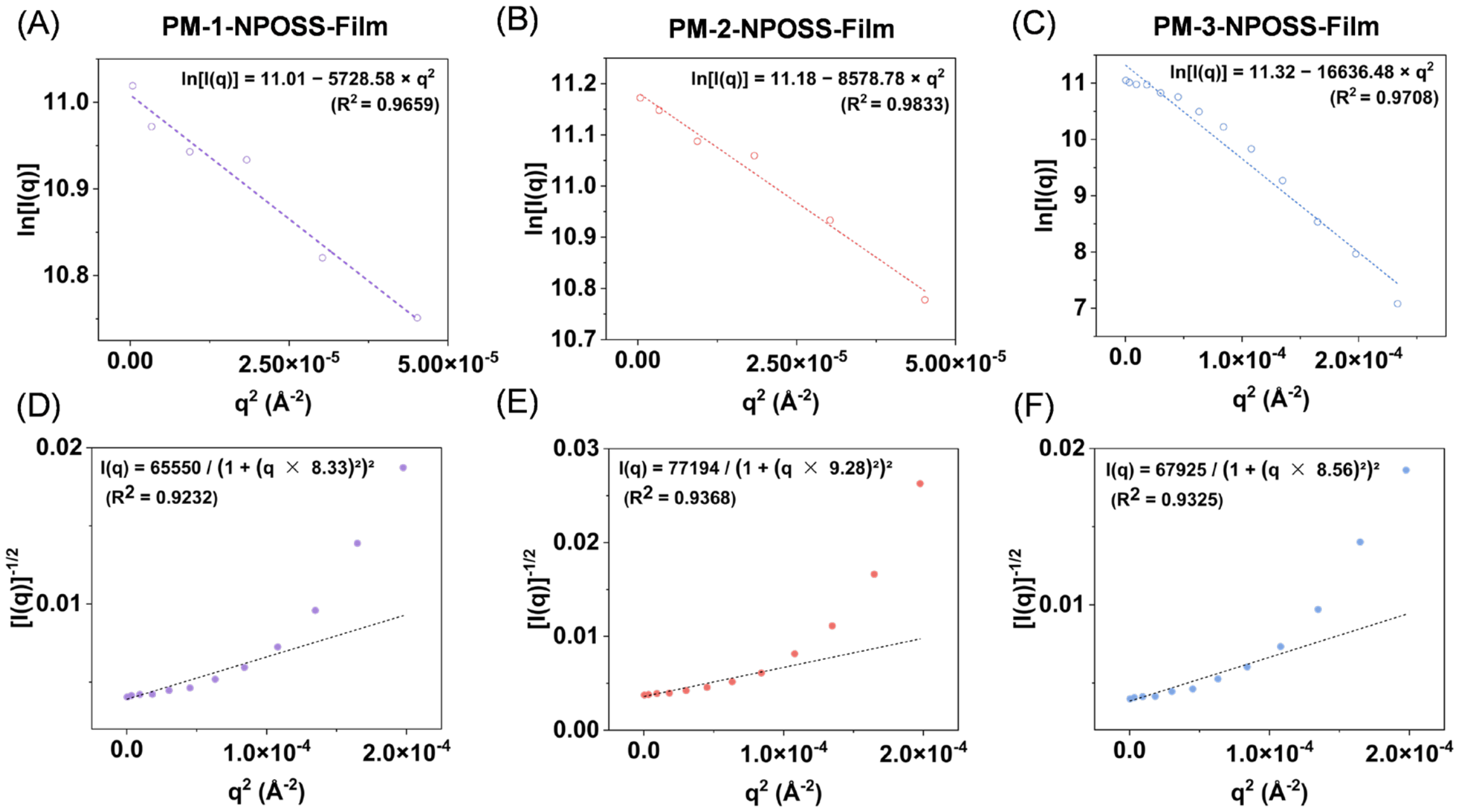

Figure S5. Guinier and Debye–Bueche Analyses of PM-1-NPOSS-Film, PM-2-NPOSS-Film and PM-3-NPOSS-Film.
(A)-(C) Guinier plots (ln I vs q^2) with linear fits in the low-q region of PM-1-NPOSS-Film (A), PM-2-NPOSS-Film (B) and PM-3-NPOSS-Film (C).
(D)-(F) Debye–Bueche plots ($I^{-1/2}$ vs q^2) with linear fits in the low-q region of PM-1-NPOSS-Film (D), PM-2-NPOSS-Film (E) and PM-3-NPOSS-Film (F).

S6. Estimated Protonation State of NPOSS

Table S4. Chloride content and estimated protonation state of as-isolated NPOSS.

Entry	Estimated Cl^- counterions per POSS cage [a]	Estimated protonated amine fraction / %
NPOSS	6.2	77.4

[a] The POSS cage in this context is a T8 structure, containing eight silicon vertices.

S7. Supplementary Characterization Statistics of PM-3-Latex Coatings

Table S5. Dry-film thicknesses of coatings.

Entry	Coating thickness / μm
PM-3-NPOSS-Coating	11.5 ± 0.6 [a)]
PM-3-ATPE-Coating	11.7 ± 2.7

[a)] Values are presented as mean ± SD from three separately coated panels (n = 3). Five positions were measured and averaged for each panel. Data were reported to three significant figures according to measurement precision.

Table S6. Optical transmittance of coatings.

Entry	Optical transmittance [a)] / %		
	UV	IR	VL
PM-3-NPOSS-Coating	82.6 ± 2.3	86.5 ± 1.8	89.6 ± 0.6
PM-3-ATPE-Coating	84.1 ± 1.2	87.5 ± 1.9	90.3 ± 0.1
Without Coating	90.0 ± 0.1	89.2 ± 0.3	91.6 ± 0.2

[a)] Values are presented as mean ± SD from three separately coated glass panels per formulation (n = 3). Three positions were measured on each panel and averaged to obtain one panel-level value. UV transmittance was measured at a peak wavelength of 365 nm, IR transmittance at 940 nm, and visible-light transmittance (VL) over 380–760 nm according to the CIE photopic luminosity function.

S8. Pot Life Measurement of the PM-3/NPOSS Formulation

The pot life was defined as the minimum period after mixing during which the formulation could still form continuous and transparent films without significant defects.The pot life of the PM-3/NPOSS formulation was evaluated following the general principles of ISO 9514. The NPOSS aqueous solution (31.60 mg/mL) was mixed with the PM-3 latex at an amine-to-epoxy equivalent ratio of 2:1 and immediately placed at room temperature. At predetermined time intervals, an aliquot of the mixed formulation was withdrawn and characterized as follows.

The formulation was applied onto circular glass panels (5 cm × 5 cm × 2 mm) using a 50 μm wire-wound drawdown bar to prepare wet films (wet-film thickness: 50 μm). After drying and curing, the film continuity and transparency were visually evaluated.

Pencil hardness was determined on the same glass panels following ISO 15184. Three separately coated panels were evaluated for each time point (n = 3).

The fineness of the formulation was measured using a fineness-of-grind gauge (Hegman gauge) following ISO 1524 (n = 1).

The formulation was applied onto black plastic panels (21 cm × 29.7 cm × 0.5 mm) using a wet film applicator with a controlled wet-film thickness of 50 μm. After drying and curing, the 60° gloss value of the coatings was measured using a gloss meter, following ISO 2813. Three positions were measured on each panel and averaged to obtain one panel-level value; three separately coated panels were evaluated for each time point (n = 3), and the results are reported as mean ± SD.

Table S7. Summary of pot life evaluation results.

Entry	Storage Time after Mixing / h	Fineness of Grind / μm	60° Gloss / GU	Pencil hardness
1	0	≤ 5 [a)]	84.1 ± 0.7	2H [b)]
2	1	≤ 5	83.4 ± 0.7	2H
3	2	≤ 5	82.4 ± 0.2	2H
4	3	≤ 5	82.6 ± 0.6	2H
5	4	≤ 5	82.9 ± 0.6	2H
6	6	≤ 5	81.9 ± 0.4	2H
7	9	≤ 5	83.0 ± 0.4	2H
8	12	≤ 5	82.6 ± 1.1	2H

a) Fineness of grind was reported as ≤ 5 μm when no visible agglomerates were observed by visual inspection (ISO 1524).

b) Pencil hardness was reported as the consistent rating obtained from three parallel measurements; the lowest rating was reported when inconsistencies occurred (ISO 15184).

(A)

(B)

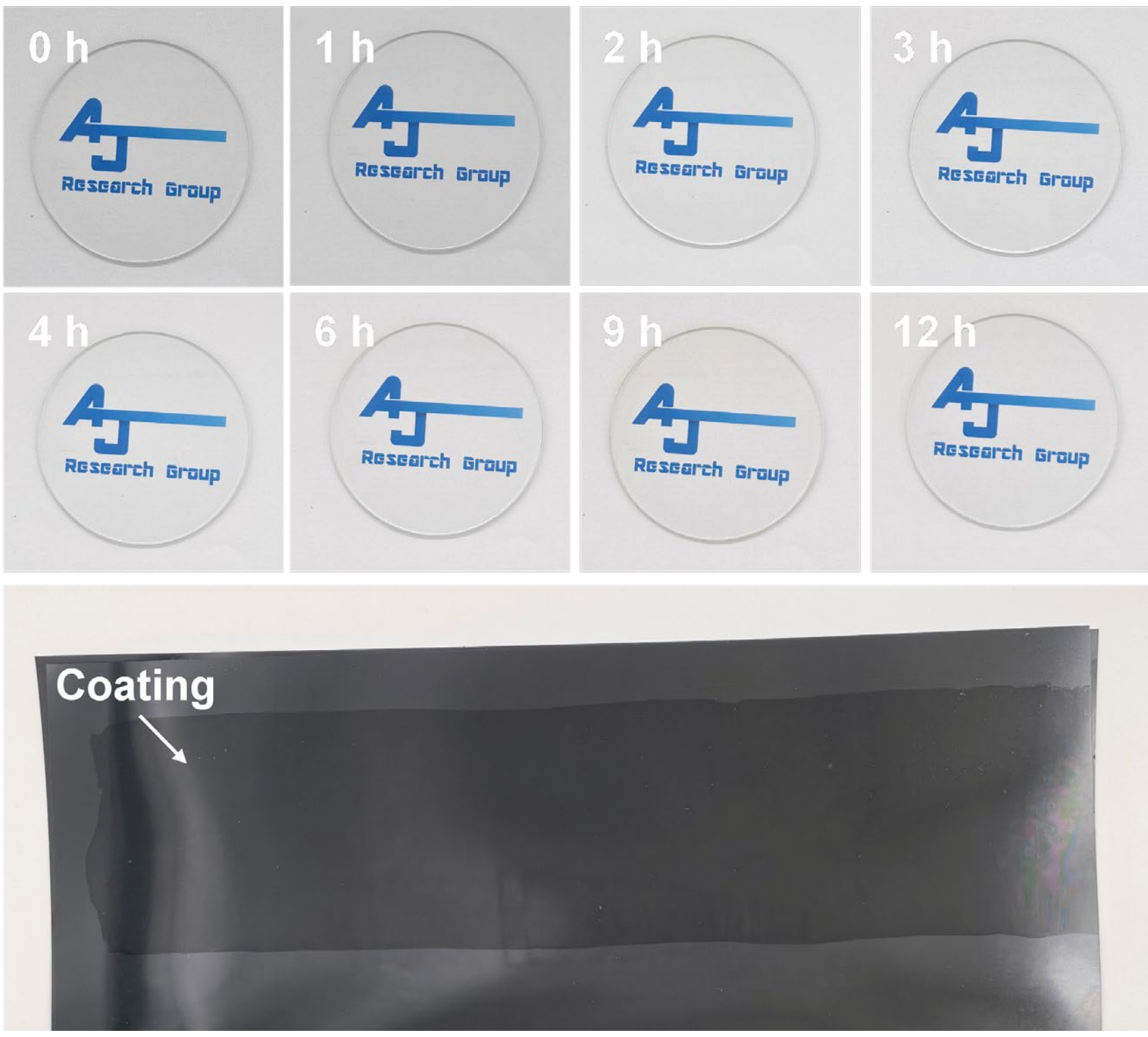

Figure S6. Pot life evaluation of PM-3/NPOSS formulation.
(A) Photographs of coatings drawn down on glass panels at different time intervals after mixing (0 – 12 h).
(B) Representative photographs of coatings applied on black plastic panels.

S9. Thermal Cycling Test of Coatings

Thermal cycling tests were performed following the general principles of ASTM D6944. PM-3-NPOSS-Coating and PM-3-ATPE-Coating were applied onto standard aluminum panels (70 mm × 150 mm × 0.5 mm) and cured under the same conditions described in Section 4.5.1. The coated panels were subjected to 18 cycles from −20 °C to 60 °C, with 2 h dwell at each temperature and rapid transitions between the two extremes. After thermal cycling, the panels were conditioned at room temperature for 24 h before evaluation.

The adhesion of the coatings was evaluated by the cross-cut test according to ISO 2409. Pencil hardness was determined following ISO 15184. The surface appearance (blistering, cracking, delamination, and gloss loss) was visually inspected under standardized lighting conditions. All tests were performed on three separately coated panels per formulation (n = 3).

Table S8. Thermal cycling resistance of PM-3-based coatings.

Entry	Sample	Adhesion rating before test	Adhesion rating after test [a]	Pencil hardness before test	Pencil hardness after test
1	PM-3-NPOSS-Coating	Class 0	Class 0	2H	2H
2	PM-3-ATPE-Coating	Class 1	Class 4	HB	HB

[a] Values were reported as the rating consistently obtained across three panels; the lowest rating was reported when inconsistencies occurred. Thermal cycling was performed following the general principles of ASTM D6944 (−20 °C to 60 °C, 18 cycles, 2 h dwell at each temperature). Adhesion and pencil hardness were evaluated after 24 h recovery at room temperature.

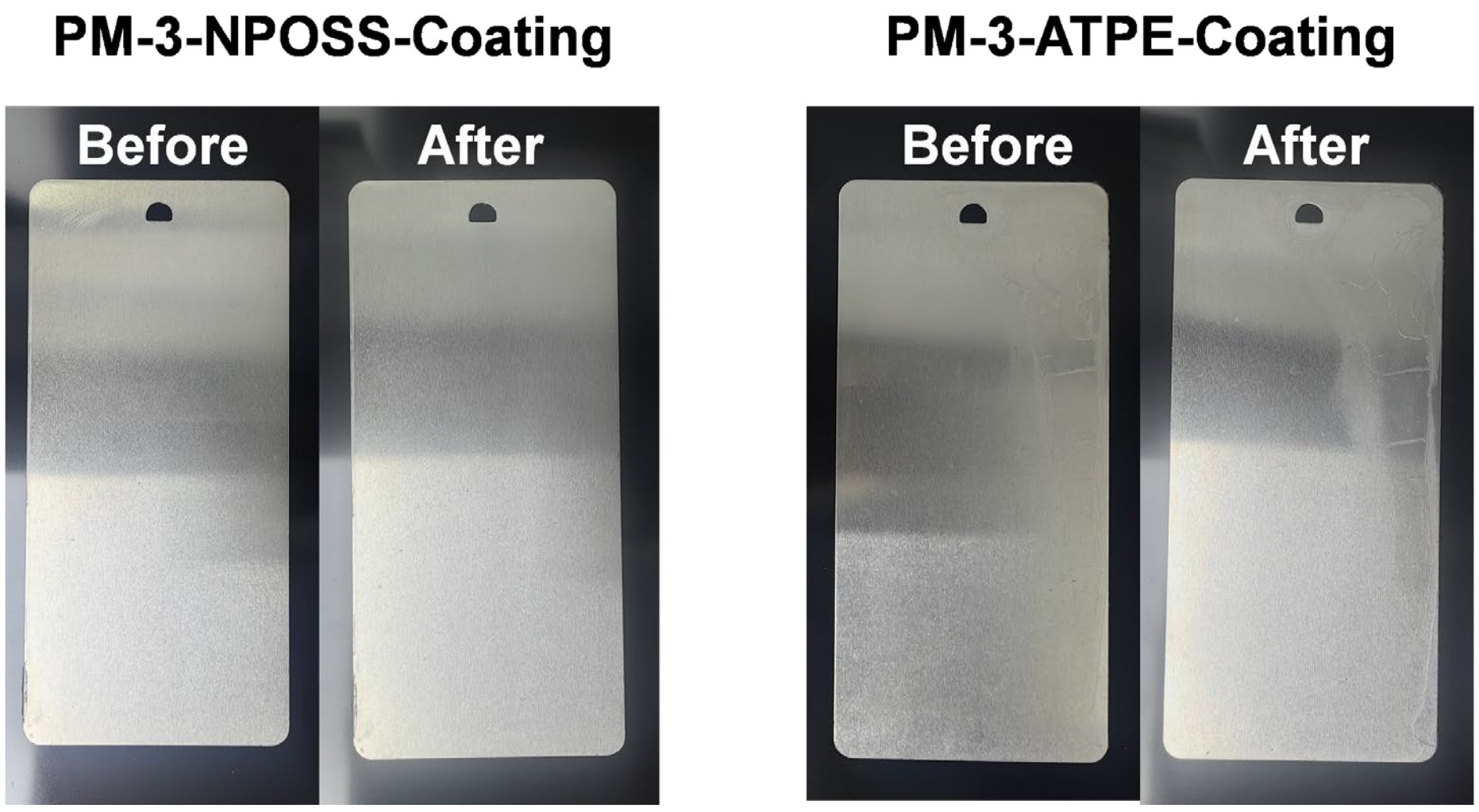

Figure S7. Photographs of coatings before and after thermal cycling.

S10. NMR Spectra of NPOSS

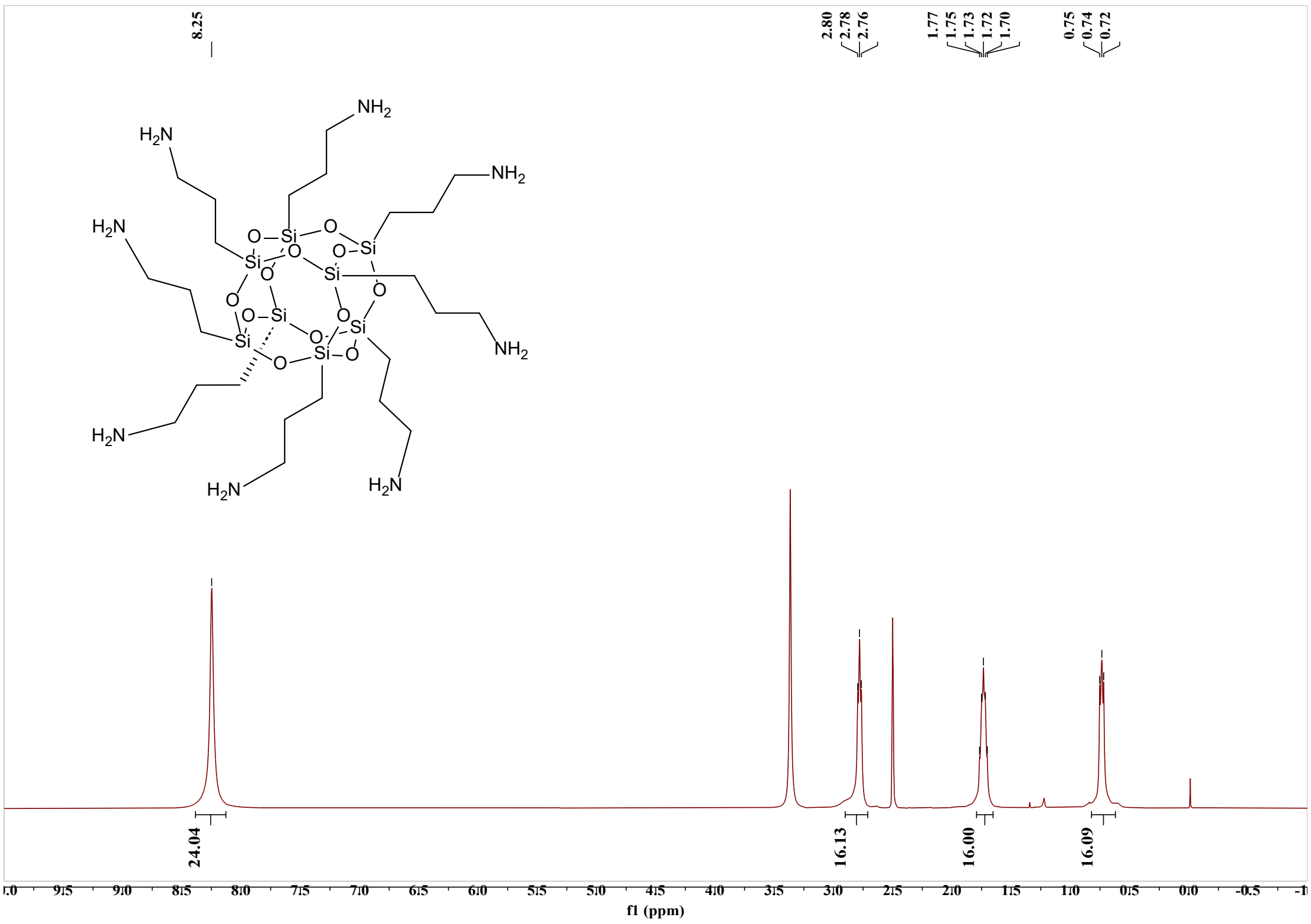

Figure S8. ^{1}H NMR spectrum of NPOSS (500 MHz, DMSO-d_6).

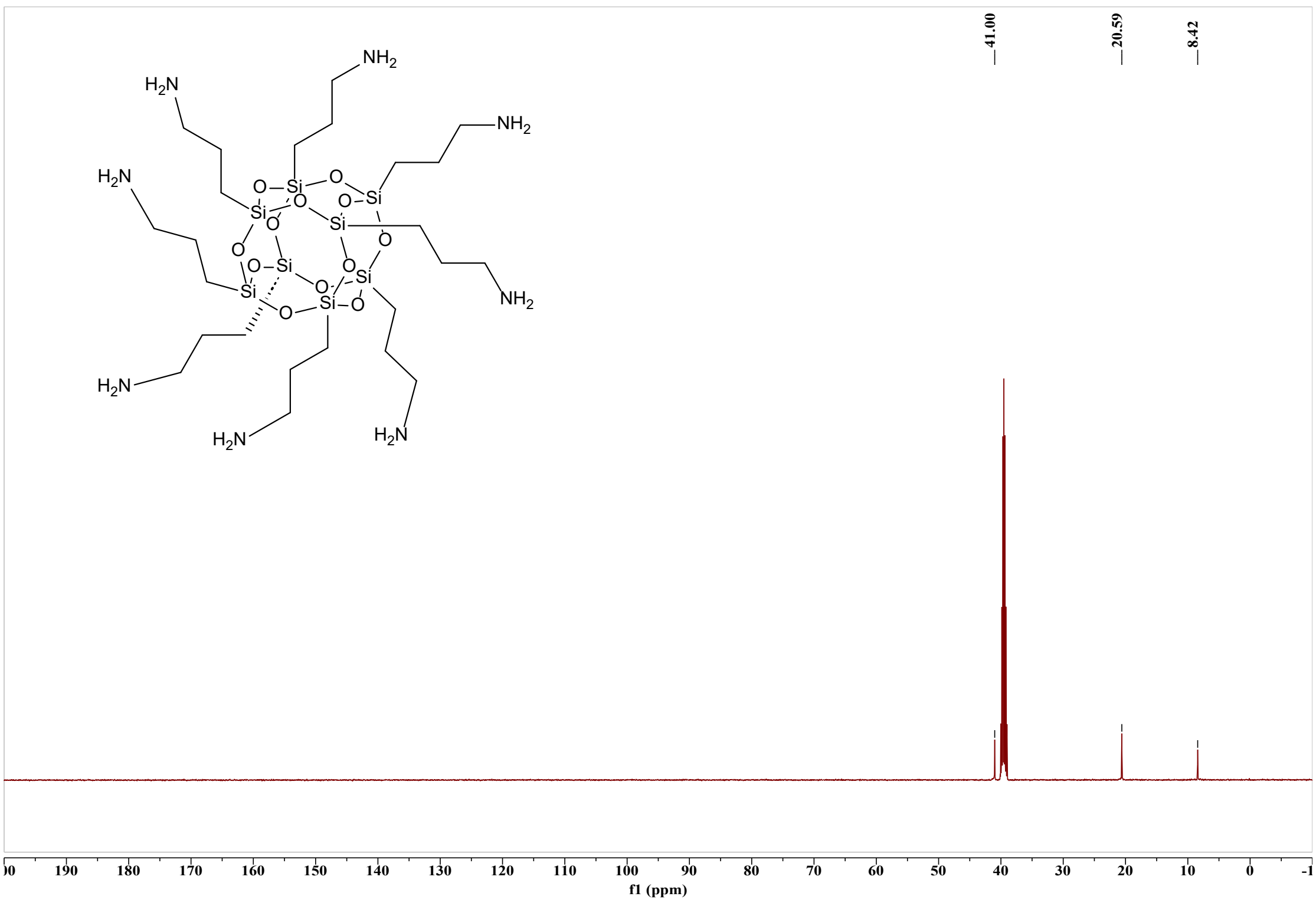

Figure S9. $^{13}C\{^{1}H\}$ NMR spectrum of NPOSS (126 MHz, DMSO-d_6).

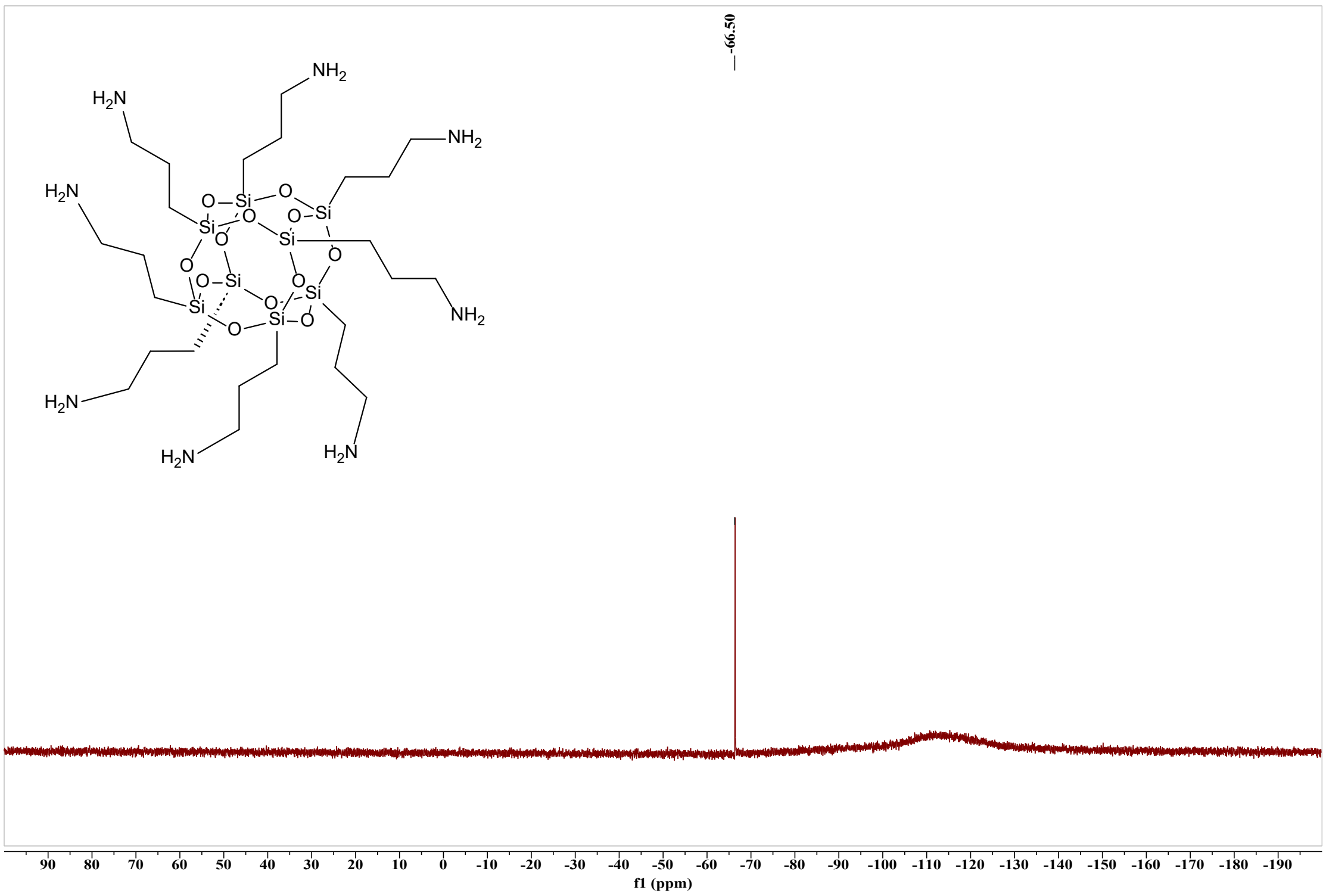

Figure S10. $^{29}Si\{^1H\}$ NMR spectrum of NPOSS (79 MHz, DMSO-d_6).

S11. AFM-IR Detection Area for Cross-Section of PM-1-NPOSS-Film

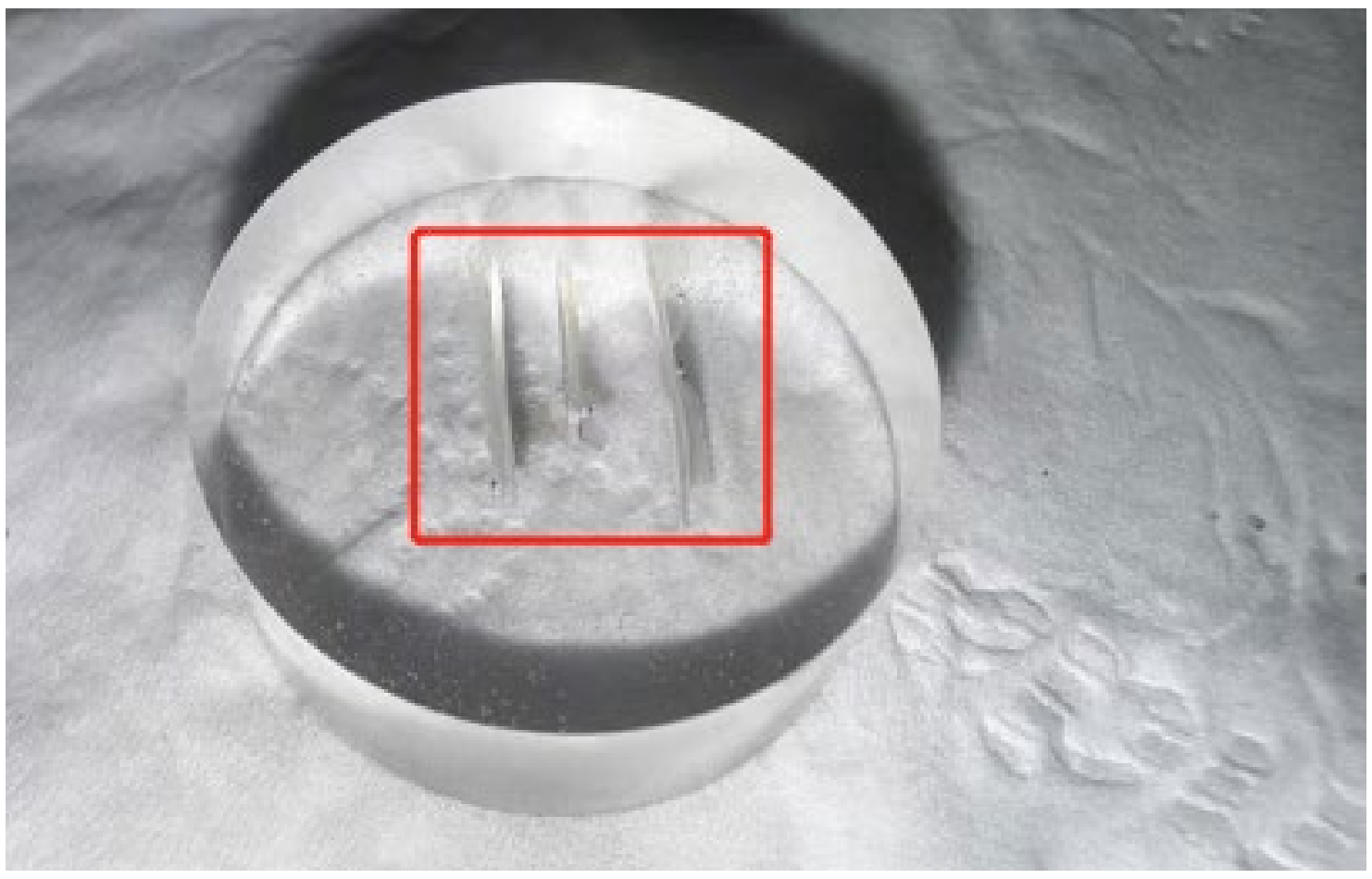

Figure S11. Macroscopic location from which the PM-1-NPOSS-Film cross-section was selected for AFM-IR analysis.
The film cross-section was embedded in epoxy resin by cold mounting and subsequently polished to obtain a flat surface. The AFM-IR detection area was located at the cross-section of the mounted film. The analyzed region corresponds to the cross-sectional surface of the film.

S12. Supplementary Statistics of SEM-EDS for PM-1-NPOSS-Film

Table S9. SEM-EDS elemental composition of PM-1-NPOSS-Film.

Chemical elements	Type of lines	wt %	At% [a)]
C	K line system	77.3 ± 0.2	83.2
O	K line system	18.3 ± 0.2	14.8
Si	K line system	4.50 ± 0.04	2.07
Total amount		100.0	100.0

[a)] All elemental concentrations were normalized to 100 wt%. Accordingly, the reported values are semi-quantitative and are used primarily to assess the absence of obvious micrometer-scale Si-rich aggregation rather than to determine absolute elemental composition.

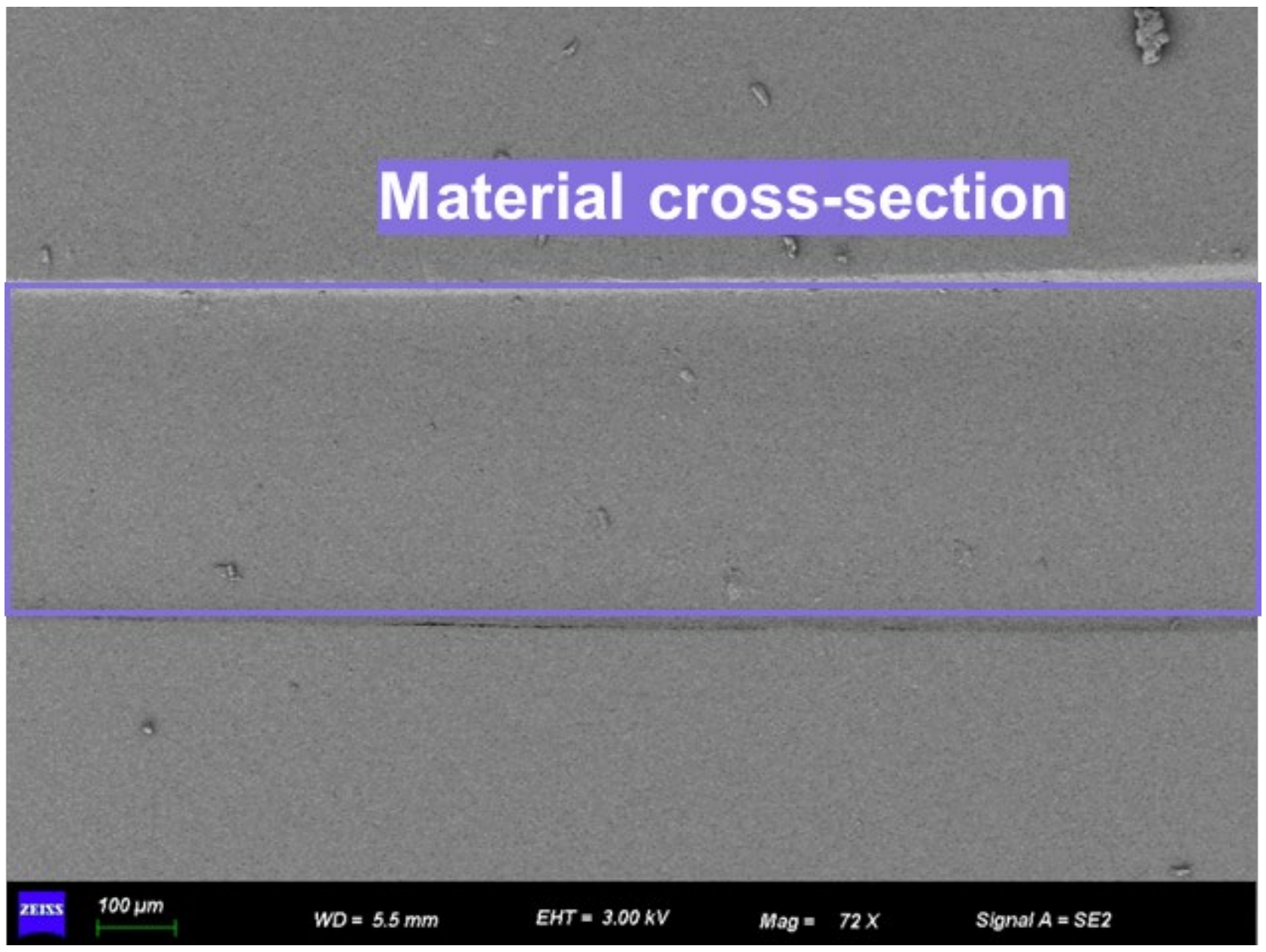

Figure S12. SEM-EDS-Mapping area on the PM-1-NPOSS-Film cross-section.
